# The origins of multi-cropping agriculture in Southwestern China: Archaeobotanical insights from third to first millennium B.C. Yunnan

**DOI:** 10.1007/s41826-022-00052-2

**Published:** 2022-05-25

**Authors:** Rita Dal Martello

**Affiliations:** Department of Archaeology, Max Planck Institute for the Science of Human History, Kahlaische Strasse 10, 07745 Jena, Germany

**Keywords:** Yunnan, Southwest China, Archaeobotany, Agriculture, Multi-cropping

## Abstract

Yunnan’s location at the crossroad of temperate China, Northeast India and tropical mainland Southeast Asia makes it a pivotal area for the understanding of early cultural contacts and agricultural spread between these ecologically diverse regions. This paper evaluates current evidence relating to the emergence of the first agricultural systems in Yunnan. It also reviews previous theories on agricultural dispersal to Yunnan, including whether Austroasiatic speakers were responsible for the spread of rice from Yunnan to mainland Southeast Asia, and builds a new framework that allows to tie agricultural development in the region into broader patterns of early migration and exchange networks. Archaeobotanical remains attest to an initial spread of rice and millet from Central China into Yunnan in the third millennium B.C. and the establishment of a mixed-crop economy; the introduction of wheat and barley in the second millennium B.C. allowed for increased diversification of the agricultural system, with a two-season intensification trend in the late first millennium B.C. Differences in early rice cultivation ecologies between Yunnan and mainland Southeast Asia suggest that Yunnan rice farmers may not have had a primary role in the southern dispersal of rice, however, more data is needed to fully clarify the source and development of dryland cultivation of rice in mainland Southeast Asia.

**Supplementary Information:**

The online version contains supplementary material available at 10.1007/s41826-022-00052-2.

## Introduction

There remains a dearth of data relating to the early agricultural practices of the remote southwestern Chinese province of Yunnan. Systematic archaeobotanical investigation in the province began a little more than 10 years ago, and archaeological excavations are primarily linked to rescue campaigns, due to rapid infrastructure development. Archaeological research in Yunnan has, to some extent, suffered from the heavy focus within Chinese Archaeology on the Central Plains, traditionally seen as the “cradle of Chinese civilisation” (e.g. Shelach-Lavi 2015; Von Falkenhausen 1993; Chang 1964). On the other hand, the mountainous and undeveloped landscape of Yunnan also contributed to the lack of archaeological research. Nevertheless, archaeobotanical research is being increasingly undertaken during archaeological excavations in Yunnan (Li Xiaorui, personal comment 2018; Li 2016), and numerous archaeobotanical reports are being published as result (i.e. Li and Liu 2016; Li et al. 2016; Dal Martello et al. 2018; Yao et al. 2020; Dal Martello et al. 2021), clarifying the role of farming in the paleoeconomy, and contributing to create a growing interest in the prehistory of Yunnan. In this paper, I review the recently accumulated archaeobotanical evidence and evaluate former theories of agricultural spread within Yunnan, delineating the development of agricultural practices in the province between the third and first millennia B.C. and thus providing a new chronological framework from which to discuss the early social development of the region.

## Geography and climate of Yunnan

**Fig. 1:**
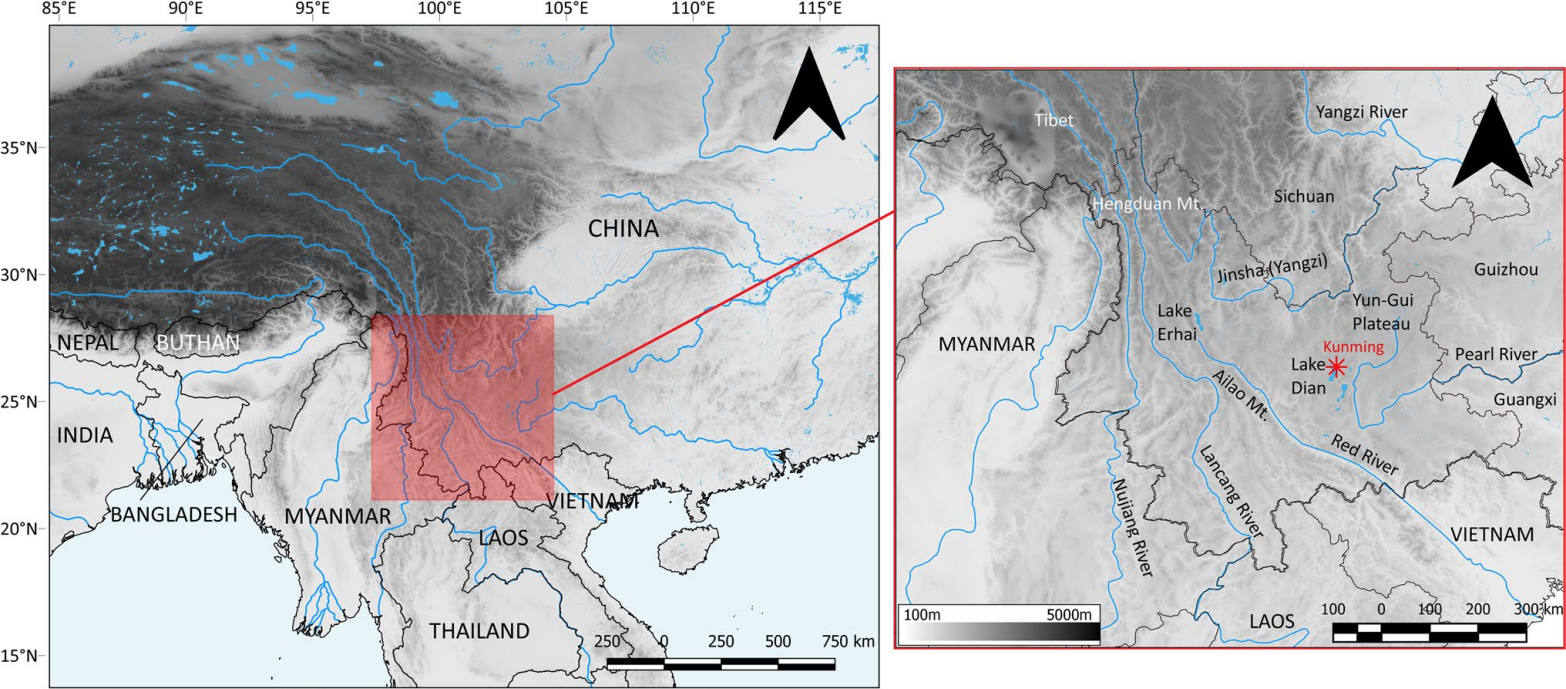
Location of Yunnan, with main mountain chains, rivers and lakes. Made with QGIS 3.16.1

Yunnan province is located in southwestern China; it borders the Chinese provinces of Tibet, Sichuan, Guizhou, and Guangxi, and the Southeast Asian countries of Vietnam, Laos, and Myanmar. Mountains cover 94% of the province’s surface, stretching north to south from over 7,000masl at Gonggashan, the highest peak in the region, to roughly 70masl at the bay of the Yuanjing River, close to the Vietnam border (Fig. 1; Tang 2015; Zhu 1985). The mountains are crisscrossed by a rich hydrological network. Three major Asian rivers run through the province, the Yangzi, the Lancang (known as Mekong in English), and the Nujiang (known as Salween in English), and the sixth largest lake of China, the Lake Dian, sits at the middle of the Yungui Plateau in central Yunnan (Fig. 1), with numerous smaller tributaries creating deep riparian valleys. Although affected by both the Pacific (Southeastern) and Indian (Southwestern) monsoons, Yunnan’s climate is mild thanks to three geophysical barriers, including: 1) the Tibetan Plateau easing the effects of the Siberian northerlies; 2) the Hengduan Mountains (22–32.05 N; 97–103 E) further mitigating the Southwestern monsoon; 3) the Ailao Mountains (23.49 N, 101.33 E) reducing the effect of the Southeastern monsoon (Fig. 1; Tang 2015). The topography of the region allows for a year-round mild and warm climate, with short and dry winters, and hot and wet summers (Kottek et al. 2006; Tang 2015).

The subtropical evergreen broadleaved forest is characterised by a so-called “biogeographical vertical zonation” that mirrors the altitudinal gradient both north to south, but especially creates a sequence of vegetation belts (tropical, subtropical, temperate, subalpine, and alpine) from the lowlands to the highlands within each valley (Tang 2015). Due to this peculiar and highly diverse landscape, Yunnan possesses the richest plant biodiversity in modern China, counting 14,822 species of native seed plants, corresponding to roughly 49% of the total seed plants in the country (Myers 1988; Walker 1986; eFloras 2008).

The biodiversity correlates with highly favourable conditions for agricultural production, with modern records indicating harvests of up to three crops per year in some parts of the province, including double or triple cropping of irrigated rice (*Oryza sativa*) in the lowlands, and double cropping of rice and winter free-threshing wheat (*Triticum aestivum*) above 1,000 m (Zhao 1994: 38; NBS 2019). Han Dynasty records (206 B.C.- 220 A.D.) describe irrigated rice fields in lowland central Yunnan from at least the first century A.D. (Yao et al. 2015) and historically, lowlands irrigated rice cultivation has been linked with the Dai ethnic population (Bray 1986).

### Ancient environment in Yunnan

Paleoclimatic records show that Yunnan has maintained a roughly stable Holocene vegetation community since the late second millennium B.C. (i.e. Hillman et al. 2017; Jones et al. 2012; Shen et al. 2005, 2006; Yu et al. 2000; Hodell et al. 1999; Whitmore et al. 1994; Brenner et al. 1991; Fang 1991; Long et al. 1991; Walker 1986). Lake sediments and pollen records indicate that early Holocene temperatures were 2/3˚C higher than at present and the monsoonal intensity was stronger than today. Between 3500 and 1500 B.C., the monsoonal intensity decreased, temperatures cooled, and in turn, the evergreen broadleaf forests declined (Chen et al. 2014). Lake sediments and pollen records from Lake Erhai, in northwestern Yunnan, indicate an increased presence of “disturbance taxa” (i.e. *Artemisia*, Pinus, Chenopodiaceae, Poaceae), which have been interpreted as a sign of human activity, possibly linked with agricultural practices and a more open landscape (Dearing et al. 2008; Shen et al. 2005). During the second millennium B.C., the weakening of the monsoon accelerated, with a sharp drop event happening at around 1500 B.C. (Dykoski et al. 2005), which brought the climatic conditions close to those of present-day.

## Sites and chronology

**Fig. 2:**
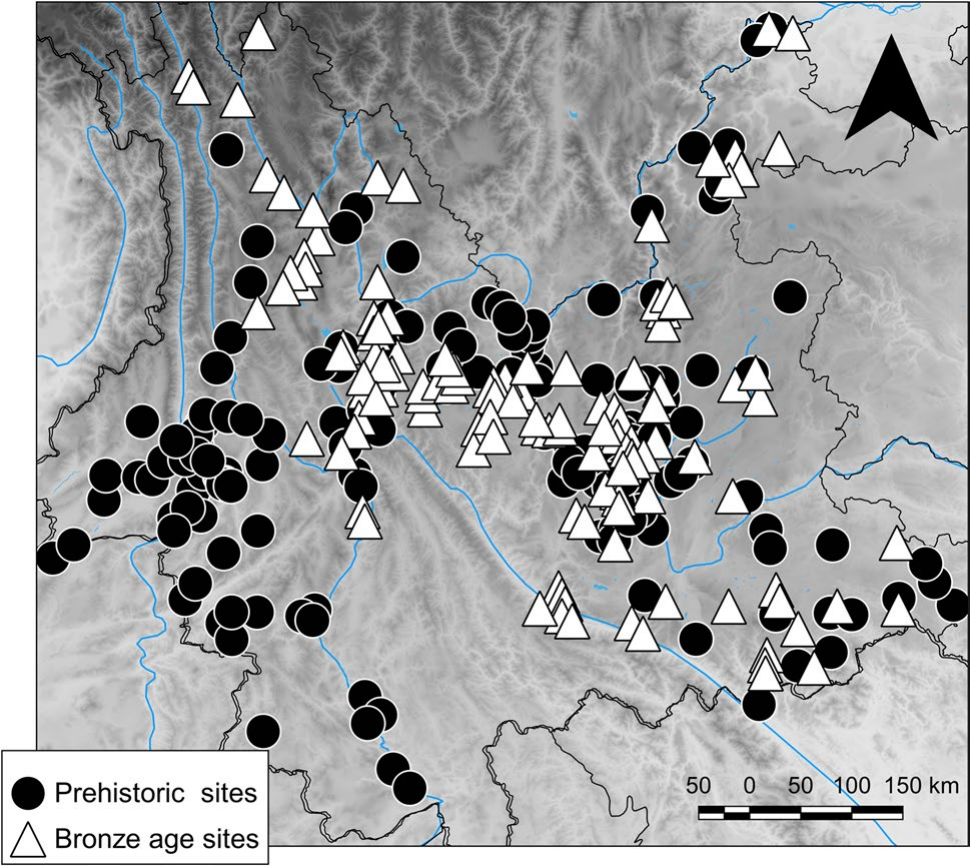
Distribution of known prehistoric (including Palaeolithic and Neolithic sites) and Bronze Age sites, as reported in the Atlas of Cultural Relics: Yunnan Volume (BNCR 2001; Remade from Hosner et al. 2016). Made with QGIS 3.16.1

The Atlas of Cultural Relics compiled by the Bureau of National Cultural Relics (BNCR 2001; Hosner et al. 2016) indicates Yunnan as one of the Chinese provinces with the lowest reported prehistorical site density (less than 1:1000km^2^). Little over 500 total prehistoric sites are reported in the Atlas and divided into so-called Palaeolithic sites (*jiushiqi shidai*), indicating archaeological sites with no reported presence of domesticated plants and/or animals, metal objects, and pottery; Neolithic sites (*xinshiqi shidai*), with reported pottery and sometimes presumed domesticated animal bones and crops, but no metal objects; and Bronze Age sites (*qingtong shidai*) characterised by the presence of metal (bronze or copper-based) objects. Although numerous sites categorised by the Atlas as Neolithic are described as settlements and many Bronze Age sites as cemeteries, the great majority of sites is simply described as “site with evidence of” lithics, pottery or bronze objects which were found on the surface during surveys. Notably, the data presented in the Atlas is derived from non-systematic surveys and rescue excavations compiled unevenly over decades and mostly focusing on areas, such as lake and river basins, where archaeologists already expected the presence of settlements to be (Jaffe et al. 2021; Jaffe and Hein 2020). The dating of the sites, based on the cultural association of the surface findings, has also sometimes been contradicted by later systematic excavation (i.e. Liu and Sun 2009). Moreover, for the specific case of Yunnan, a variety of sites are included under the “Palaeolithic” umbrella, including early hominid sites that are dated to several hundred years ago, such as the Yuanmou Man site (i.e. Huang and Grun 1998; Yue et al. 2004), but these are not clearly indicated in the Atlas’ maps, further complicating direct inferences made from it. For these reasons, the data presented by the Atlas can provide only a partial picture of past settlement density and distribution, and although the mountainous landscape of Yunnan might have contributed to a low prehistoric occupation, the limited archaeological research in the province is more likely to account for much of the scarcity of the currently available data (Fig. 2).

Li and Hu (2009) reported that only a minority of these sites had been formally excavated until 2009 (keeping with the division outlined in BNCR 2001; 17 Palaeolithic, 32 Neolithic, and 48 Bronze Age sites) and although the total number of excavated sites has surely increased since then, only a handful of preliminary excavation reports have been published (i.e. Jiang and Wu 2020; Hu et al. 2016; Chang and Hu 2011).

The published literature dates the transition to an agricultural way of life roughly between the third and second millennium B.C. when the first settled villages appear in the province (Dal Martello et al. 2018; Li et al. 2016; Yao 2010; Zhang and Hung 2010; Li and Hu 2009). Currently, excavated settled villages are located in river and lake basins, usually dividing into open-air sites along the rivers, shell-midden sites around the lakes, and a few cave sites on the rugged western side of the province.

**Fig. 3:**
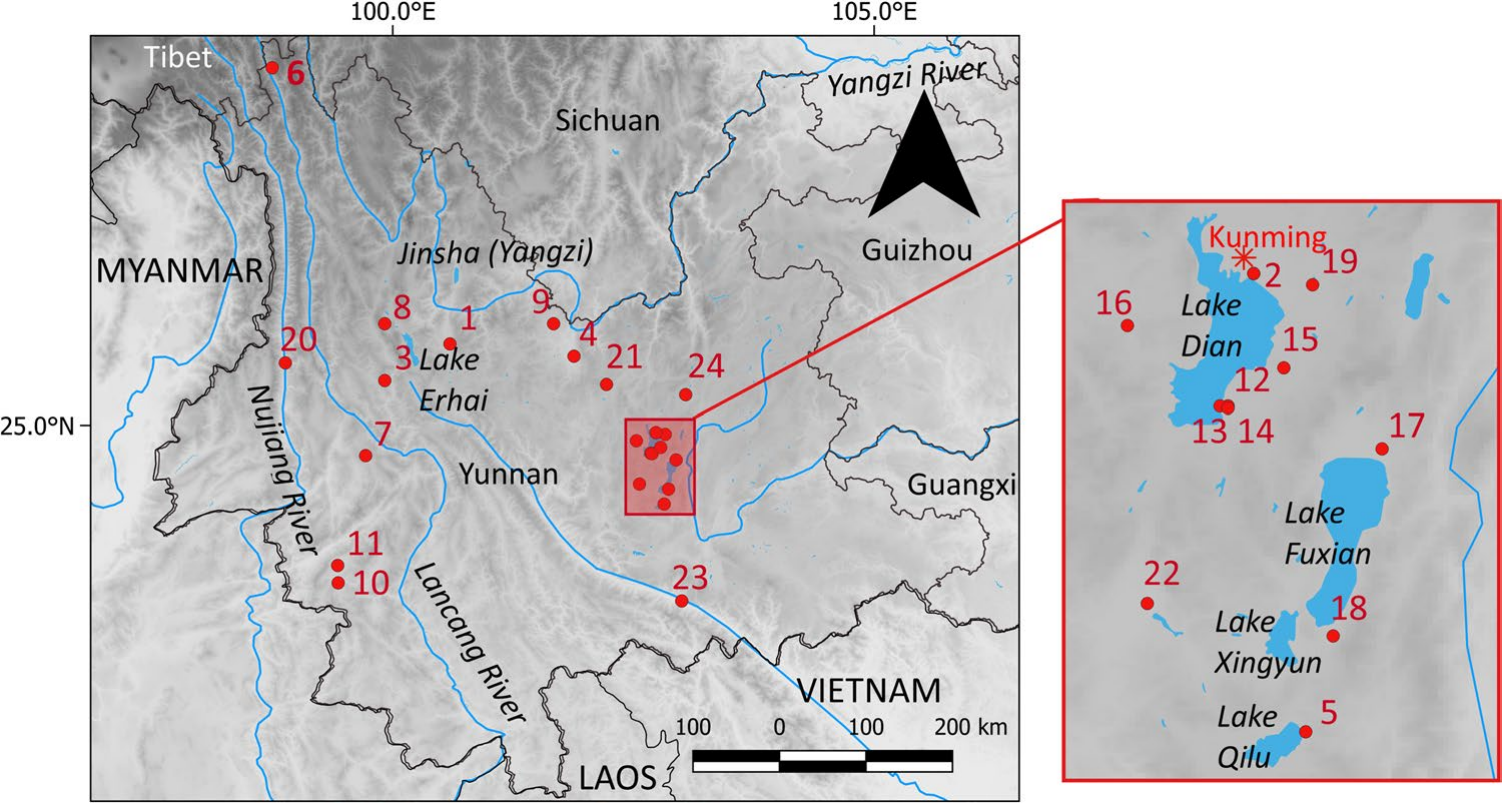
Location of sites with reports of ancient plant remains in Yunnan, listed in Table 1: 1. Baiyangcun; 2. Haidong; 3. Xinguang; 4. Dadunzi; 5. Xingyi; 6. Zongzan; 7. Yingpanshan; 8. Haimenkou; 9. Mopandi; 10. Shifodong; 11. Nanbiqiao; 12. Shizhaishan; 13. Hebosuo; 14. Shangxihe; 15. Anjiang; 16. Dayingzhuang; 17. Xueshan; 18. Guangfentou; 19. Xiaogucheng; 20. Shilinggang; 21. Yubeidi; 22. Jinlianshan; 23. Mayutian; 24. Qujing Dongjia. Made with QGIS 3.16.1

Only two sites from the third millennium B.C. have been dated through AMS radiocarbon dating (Tables 1, S1; Fig. 3): Baiyangcun (2650–1690 cal B.C.; Dal Martello 2020; Dal Martello et al. 2018); and Dadunzi (2200–1650 cal B.C.; Jin et al. 2014). These two sites are both located in the Jinsha basin, an affluent of the Yangzi River, in northwestern Yunnan; they are characterized by wattle and daub houses and rectangular shaft pit burials. A small number of stone cist burials were recovered from Dadunzi, and both sites share similarities in ceramic remains (Table S2). The pottery vessels retrieved at both sites constitute, for the most part, coarse greyish temper, decorated with the so-called incised/impressed ceramic style, characterised by geometrical and dotted designs (Rispoli 2007). *Guan* jars with flat/round bases, ovoid bodies, and outward protruding openings, make up the majority of the ceramic vessels retrieved (YPM 1977; YPM 1981). This type of *guan* jar is suitable for cooking and serving liquids and/or semi-liquid substances. In the later phase of occupation, *guan* jars increase in number and pouring vessels, with double handles, high necks, and/or spouts, appear at both Baiyangcun and Dadunzi. Traces of red paints have been reported on the early ceramic remains from the site of Xinguang (YPICRA 2002). Shell-midden sites from central Yunnan show a distinctive ceramic tradition of corded wares (Yao 2010).

**Table 1:** Comparison of sites in Yunnan with reported ancient plant remains. See Table S2 for a full comparison of cultural material and environmental remains

Site	Location	Exc. Date	Chronology	Main archaeobotanical remains	References
1. Baiyangcun 白羊村	Middle Jinsha, Binchuan county	1973/74 2013/2014	AMS 2650–1690 cal BC	*Oryza sativa* *Setaria italica* *Panicum miliaceum* *Echinochloa* sp*.* *Glycine soja* *Vigna* sp. *Cajanus* sp. *Cucumis* sp. *Euryale ferox* *Vitis* sp.	YPM 1981; Dal Martello et al. 2018; Dal Martello 2020
2. Haidong 海东	Qilu Lake, Tonghai county	1988/89	c. 2500- 1750 BC	Rice [hand- picked]	He 1990; Xiao 2001; Zhang and Hung 2010; Yao 2010
3. Xinguang 新光	Upper Lancang, Yongping county	1993/94	c. 2500–1750 BC	Charred rice grains from G3 [hand-picked]	YPICRA 2002; Yao 2010
4. Dadunzi 大墩子	Middle Jinsha, Yuanmou county	1972/73 1999/2010	AMS 2200–1650 cal BC	*Oryza sativa* *Setaria italica* *Panicum miliaceum* *Vigna* sp. Cucurbitaceae	Jin et al. 2014
5. Xingyi 兴义	Qilu Lake, Tonghai county (Kunming)	2015/16	c. 2000–0 BC	Acorns, *Oryza* sp. (unpubl.)	YPICRA 2017
6. Zongzan 宗咱	Upper Lancang, Weixi county	2013	c. 2000- 200 BC	Buckwheat?	Li 2016 Chen et al. 2019
7. Yingpanshan 营盘山	Between Lancang- Nujiang, Changning county	1990	c. 1800 BC	Rice [hand- picked]	Xiang et al. 2015
8. Haimenkou 海门口	Middle Jinsha, Jianchuan county	1957 1978 2008	AMS 1600–400 cal BC	*Oryza sativa* *Setaria italica* *Panicum miliaceum* *Chenopodium* sp. *Triticum aestivum* *Hordeum vulgare* *Fagopyrum* cf *esculentum* *Cannabis* sp. *Prunus* cf *persica* *Prunus* cf *armeniaca* *Quercus* sp.	YPM 1958; Xue 2010; Jin 2013; Li and Min 2014; Dal Martello 2020
9. Mopandi 磨盘地	Middle Jinsha, Yongren county	1983	c. 1400 BC	Rice	YPCRA 2003; Zhao 2003
10. Shifodong 石佛洞	Middle Lancang, Gengma county	1982 2003	c. 1400–1100 BC	*Oryza sativa* *Setaria italica* *Chenopodium* sp. *Tamarindus* cf *indica* Indet. tree legume	Kan 1983; Liu and Dai 2008; Yao 2010; Zhao 2010
11. Nanbiqiao 南碧桥	Lower Lancang	1982	c. 1250–970 BC	Rice [hand-picked]	Kan 1983; An 1999
12. Shizhaishan 石寨山	Dian Lake, Jinning county	1953 1955 1958 1960	AMS 779–488 cal BC	*Triticum aestivum* *Oryza sativa* *Setaria italica*	YPM 1963; Yao and Jiang 2012
13. Hebosuo 河泊所	Dian Lake, Jinning county	2014	AMS 1186–945 cal BC/ 789–674 cal BC	*Oryza sativa* *Triticum aestivum* *Setaria italica* *Panicum miliaceum* *Glycine max*	Yao et al. 2020; Yang 2016; Yao et al. 2015; Yao and Jiang 2012
14. Shangxihe 上西河	Dian Lake, Jinning county	2014	AMS 1212–209 cal BC	*Oryza sativa* *Triticum aestivum*	Yao et al. 2020; YPICRA and Chicago 2019
15. Anjiang	Dian Lake, Jinning county	2008 2010/11	AMS 770- 430 cal BC	*Oryza sativa* *Triticum aestivum* *Hordeum vulgare* *Setaria italica* *Panicum miliaceum* *Chenopodium* sp*.*	Yao et al. 2015
16. Dayingzhuang 大营庄	Dian Lake, Kunming	2017	AMS 750–390 cal BC	*Oryza sativa* *Setaria italica* *Triticum aestivum* *Hordeum vulgare* *Chenopodium* sp*.* *Zantoxhylum* sp.	Dal Martello 2020; Dal Martello et al. 2021
17. Xueshan 学山	Dian Lake, Chengjiang county	2010	c. 700 BC- 100 AD	*Triticum aestivum* *Oryza sativa* *Setaria italica* *Panicum miliaceum* *Glycine max* *Fagopyrum* cf *Hordeum vulgare* *Zanthoxylum* sp. Fruits, Acorns	Wang 2014; Wang et al. 2019
18. Guangfentou 光坟头	Fuxian Lake, Jiangchuan county	1984 2011/12	c. 700–300 BC	*Triticum aestivum* *Oryza sativa* *Setaria italica* *Hordeum* sp *Panicum miliaceum* *Chenopodium* sp.	Li and Liu 2016
19. Xiaogucheng	Dian Lake	2008 2010/11	c. 700–300 BC	*Oryza sativa* Panicoideae	Yao et al. 2015
20. Shilinggang 石岭岗	Middle Nujiang, Lushui county	2003 2013/14	AMS 723- 339 cal BC	*Oryza sativa* *Setaria italica* [Isotopes] Mix C3-C4; Tubers, roots Acorns Palms	Li et al. 2016; Ren et al. 2017; Zhang et al. 2017
21. Yubeidi 玉碑地	Bingu River (Jinsha River), Dongchuan County	2013	c. 700–200 BC	*Oryza sativa* *Setaria italica* *Triticum aestivum* *Glycine max* *Chenopodium* sp. *Zantoxhylum* sp.	Yang 2016; Yang et al. 2020
22. Jinlianshan 金莲山	Fuxian Lake, Chengjiang county	2008/2009	c. 700 BC	[Isotopes] C3 prevalence (rice?); C4 secondary	Zhang 2011
23. Mayutian 麻玉田	Red River, Yuanjiang section	2006	c. 400–300 BC	[Isotopes] Mixed C3-C4	Xiao and Wan 2013 Zhang et al. 2014
24. Qujing Dongjia Village 曲靖董家村	Dian Basin	1982	c. 700–300 BC	Rice [hand-picked]	Li and Li 1983

Starting in the mid-second millennium B.C., bronze objects have been reported from Haimenkou (Wang 2018; Xue 2010; Liu and Chen 2012; Li and Hu 2009; Xiao 2001; YPM 1958). Sites located in the northwest of the province (Fig. 3), such as Haimenkou (1600–400 cal B.C.) and Mopandi (ca. 1400 B.C.), still show the earlier impressed/incised pottery style, characteristic of this area of Yunnan (Rispoli 2007). The ceramic repertoire at the initial phase of occupation of Haimenkou also shows similarities with the Baiyangcun and Dadunzi ceramic tradition; however, houses in this area are of the stilt type (YPM 1958; Xue 2010; Table S2). On the western border, along the Lancang Basin, two cave sites have been investigated: Shifodong (ca. 1400–1100 B.C.; Liu and Dai 2008), and Nanbiqiao (ca. 1250–970 B.C.; An 1999; Kan 1983). This ceramic assemblage presents a mixture of characteristic northwest Yunnan incised/impressed ceramic style, and Southeast Asian motifs (Rispoli et al. 2013; Liu and Dai 2008; Kan 1983). An additional site, Yingpanshan, in Changning county, between the Lancang and Nujiang Rivers, was excavated in 1990 and dated to c. 1800 B.C. (Xiang et al. 2015); two bronze knives were found by farmers and prompted the excavation of the site, however, how these related to the site stratigraphy remained unclear.

Comparatively more sites dating to the first millennium B.C. have been excavated; these mostly cluster in the Dian Basin of central Yunnan (Fig. 3), and are associated with the Dian Culture, a polity present in Central Yunnan from at least the eighth century B.C. until it was conquered by the Han in 109 B.C. (Allard 1999; Zhang 1997). Elite burials in the Dian cemeteries often contain lavish bronze drums and cowrie-shell containers, stylistically very different from the Bronze traditions of other areas in China; other small bronze objects such as small weapons, tools and personal accessories have also been found in Dian settlements. Metal composition analyses revealed that earlier objects were made of tin bronze alloys or pure copper; later objects show increasing quantities of lead (i.e. Zou et al. 2019).

## Previous theories on the origins of agriculture in Yunnan

Based on modern wild rice distribution, as well as accounts from early Chinese written records, scholars in the 1930s proposed that the centre for rice domestication was somewhere in the area encompassing modern Nepal, Assam, Myanmar, and Yunnan (Chang 1976; Li 1970; Chang 1964; Chatteerjee 1951; Ramiah 1937; Roschevicz 1931). In the 1920s and 1930s, Yunnan had the highest diversity of wild rice species, and it was thus suggested that rice originated there, following Vavilovian principles (Xu 1998; Li 1981; Chang 1976; Wang 1977; Li 1975). In this traditional model, domesticated rice then spread north to the Yellow River Basin and south to Vietnam along the Chinese Coast to the Yangzi Basin (Li 1970). Recent archaeobotanical work in China has shown that this model does not hold up, and currently, scholars argue that rice was domesticated along the middle and lower Yangzi Basin before the fifth millennium B.C. (i.e. Fuller et al. 2007;2009; 2014; 2016; Fuller and Qin 2009; Zhao 2011). The earliest clear dates for domesticated rice in the lower Yangzi Basin predate material from Yunnan by two millennia (Dal Martello et al. 2018). More recently, it has been proposed that southward movements of farmers from Central China brought agricultural practices to the province (Zhang and Hung 2010; Yao 2010). This was supported by the chronological delay in the appearance of agricultural systems in Yunnan compared to other parts of China and the fact that there is no highly visible presence of local hunter-gatherers’ in the province, which would suggest that they did not play a role in the adoption of agriculture. Zooarchaeological analyses undertaken at the seasonal site of Tangzigou (ca. 7000–6800 B.C.), located on the southern edge of the Gaoligong Mountains in western Yunnan (Jin et al. 2012), show that the occupants ate small cervids, some large cervids, and bovids, and possibly some micromammals. These studies also suggest that they were not under food resources stress (Jin et al. 2012; Jin 2010). The reconstructed abundant presence of edible acorns, *i.e. Cyclobalanopsis, Castanopsis,* and *Lithocarpus,* as well as fruits, including local varieties of *Prunus* (sensu lato) trees (Su et al. 2015), on Yunnan’s mountains during the Holocene would also have supported these early hunter-gatherer populations. The abundance of local wild resources would not have pushed local hunter-gatherers towards the adoption of agriculture, further supporting an external input for the first agricultural practices in the province (Liu and Chen 2012: 73). However, the difficulty in detecting the local hunter-gatherers’ presence might be biased by the insufficient archaeological investigation undertaken so far in Yunnan.

Researchers have also suggested that Yunnan was a corridor for agricultural spread from China to mainland Southeast Asia in the context of the diffusion of the Austroasiatic languages, and especially in connection to the Austric hypothesis (Higham 1996, 2004; Reid 1994, 1996, 1999; Blust 1996; Diffloth 1994). On the basis of “lexical agreements” (Diffloth 1994), morphological and syntactic similarities (Reid 1999, 1996), this hypothesis argued that Austroasiatic and Austronesian languages derived from a common ancestor (Schmidt 1906), named Austric, and its speakers originally lived and moved out from the *Sanjiang 三江*area between the Yangzi, Lancang (Mekong) and Nujiang (Salween) Rivers (Blust 1996), along the borders of modern-day Yunnan and Myanmar, bringing rice agriculture with them. Although Higham argued that the Austric homeland was in the Yangzi Basin, he proposed that the incised/impressed ceramic style tradition found both at early sites in Yunnan (i.e. Baiyangcun) and mainland Southeast Asia (i.e. Phung Nguyen, Samrong Sen, Ban Chiang, Non Pa Wai, and Khok Phanom Di) was an indication that Yunnan farmers moved southward along the Lancang (Mekong) and other river basins, dispersing rice agriculture with them to mainland Southeast Asia (e.g. Higham 2004, 2002).

A recent hypothesis of Sino-Tibetan origins would postulate that millet cultivation spread with speakers of these languages out of Neolithic central China into Southwest China and beyond to the Himalaya and Southeast Asia, establishing the cereal-based Neolithic of the region (Sagart et al. 2019).

An alternative linguistics theory for the origins of agriculture in Yunnan, instead, links the dispersal of rice out of this region, including into China. Van Driem (2012, 2017) has suggested that domesticators of rice could have involved Austroasiatic and early Tibeto-Burman speakers and ancestors to Austronesian all spreading from the eastern Himalayan region, from around the region of northeast India and Myanmar, with certain O-haplogroups of the Y-chromosome (Chaubey and van Driem 2020). As already noted above, this is at odds with archaeological evidence for rice domestication, but it raises the importance of improving the empirical evidence for past crops and plant use in Yunnan.

## Reviewing the archaeobotanical record

Before the turn of the twenty-first century, no systematic flotation had been employed in Yunnan, and the very few reports of ancient rice derived from handpicked grains, which were visible during excavations (i.e. He 1990; YPM 1981; Kan 1983; YPICRA 2002). In 2001 a small-scale flotation study was undertaken at the site of Mopandi (YPICRA 2003), and another in 2003 at Shifodong (Liu and Dai 2008), where a couple of selected contexts were analysed. Subsequently, more systematic sampling was undertaken during the third excavation campaign of Haimenkou, in 2008 (i.e. Xue 2010; Jin 2013; Li and Min 2014; Dal Martello 2020), and bulk soil sampling for flotation has since been increasingly included in excavations and sampling strategies across archaeological campaigns in Yunnan (Li Xiaorui, personal comment 2018; Li 2016). To date, less than a dozen prehistorical sites in Yunnan have undergone systematic environmental archaeology investigation through the analysis of ancient animals and/or plant remains (Tables 1; S2); additionally, few studies have investigated isotopes levels on human bones remains to reconstruct dietary intakes (Tables 1 and S2; Zhang et al. 2014; Zhang et al. 2017; Ren et al. 2017; Zhang 2011).

### Archaeobotanical remains from sites dating to the third millennium B.C.

**Fig. 4:**
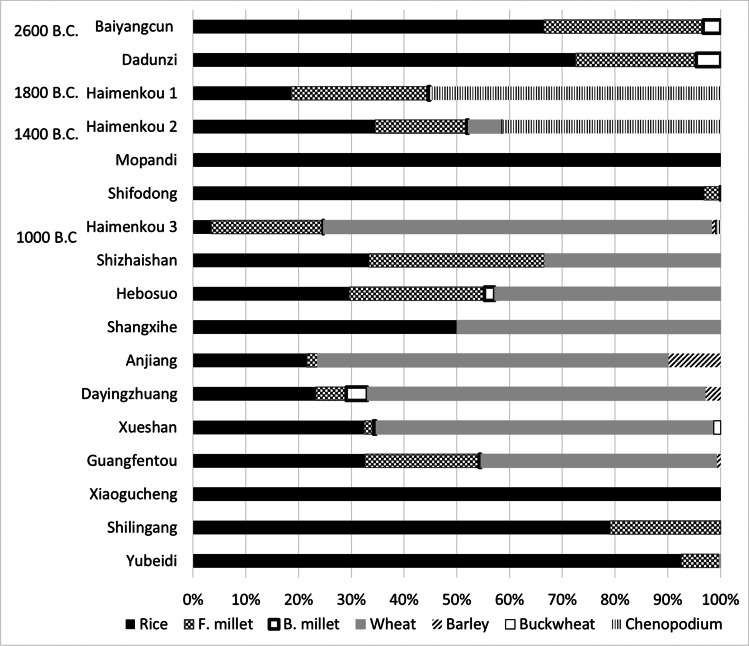
Percentage of main archaeobotanical components (rice, millets, wheat, barley, buckwheat, and *Chenopodium*) from sites in Yunnan dating between the third to the first millennium B.C. *Chenopodium* remains from Haimenkou have been included due to its interpretation as a crop at the site; although *Chenopodium* remains have been recovered in high quantities from other sites, including Guangfentou and Dayingzhuang, its status as crop or weed at those sites is undetermined and it has been decided not to include it here. Only sites where flotation was undertaken are represented in figure (data from Dal Martello 2020; Jin et al. 2014; Xue 2010; Xue et al. 2022; Zhao 2003; Zhao 2011; Yao and Jiang 2012; Yao et al. 2015; Yao et al. 2020; Dal Martello et al. 2021; Wang 2014; Li and Liu 2016; Li et al. 2016; Yang 2016). Made with Excel

At the neighbouring sites of Baiyangcun and Dadunzi, in northwestern Yunnan, archaeobotanical analyses attest to the presence of a mixed rice-millets system (Table 1, Figs. 3 and 4). Both foxtail (*Setaria italica)* and broomcorn (*Panicum miliaceum*) millet have been retrieved, although the latter in much lower quantity (Dal Martello 2020). Crops represent over 80% of the total archaeobotanical remains from these sites; pulses, wild fruits, and nuts were found across the majority of the samples, but in very low quantities (Dal Martello 2020; Jin et al. 2014). Additionally, seeds of possible field weeds, such as *Fimbristylis* sp., and *Scirpus* sp., have been reported from both sites. These same wild species have been inferred to indicate the presence of irrigated rice cultivation, from earlier sites both in the Yangzi Basin and in northern China (Fuller et al. 2016; Weisskopf et al. 2015; Deng et al. 2015; Fuller and Qin 2009). Their presence may suggest that rice at Baiyangcun and Dadunzi was cultivated using a similar semiaquatic system. Morphometric measurements of rice grains recovered at Baiyangcun show a range comparable to known domesticated rice from other archaeological sites in China (i.e. Crawford et al. 2006; Tang 1999; Li 1994; Lee and Bestel 2007; Tang et al. 2003; Fuller et al. 2014; D'Alpoim Guedes 2013; Zhao 2003; Li and Liu 2016; Pei 1998; Yang 2016; Wang 2014), and the near absence of wild-type rice spikelet bases, confirms that the rice cultivated at Baiyangcun was already domesticated. Rice grains were also handpicked at the sites of Xinguang and Haidong (YPICRA 2002; He 1990; Table 1). At Haidong and Xingyi, two shell-midden sites located in the Dian Basin, a large quantity of a lake water mollusc, *Margarya* sp., has also been reported (Fig. 3; Table S2). Preliminary archaeobotanical analysis at Xingyi revealed the presence of high quantities of acorns, suggesting people at Xingyi relied heavily on the collection of surrounding wild resources, likely from the nearby lacustrine ecosystem, and possibly practiced small-scale rice cultivation (Min Rui, personal comment 2018; YPICRA 2017).

#### Second millennium B.C.

Archaeobotanical data attests to a mixed rice-millets cultivation system at sites dating to the second millennium B.C, including Haimenkou in northwest Yunnan, and Shifodong and Yinpanshan on the Western Mountains along the Lancang and Nujiang Basins (Figs. 3 and 4; Table 1). Foxtail millet is still retrieved in much higher quantities than broomcorn millet at these sites. Rice from a partially floated context has also been reported from Mopandi, in a neighbouring valley from Haimenkou, and handpicked from Nanbiqiao, a cave site not far from Shifodong; both sites have been dated by cultural association (Kan 1983; An 1999). Of these, Haimenkou is the best investigated, with both archaeobotanical and zooarchaeological analyses undertaken (Wang 2018; Xue 2010; Jin 2013; Dal Martello 2020; Xue et al. 2022). Here, grains of wheat and barley have been reported from the second phase of occupation at the site, with wheat grains directly dated to 1440–1290 cal B.C. (Xue et al. 2022; Xue 2010; Li and Min 2014, see Table S1). This coincides stratigraphically with the appearance at the site of bronze objects, as well as an increased number of dwellings. There is also a greater abundance of stone tools connected to deforestation and woodworking, and sheep/goat bone remains (Wang 2018; Xue 2010). Of particular interest is the fact that high quantities of *Chenopodium* were found in crop rich contexts alongside remains of rice and millets. *Chenopodium* seeds constitute almost half of the recovered staple crop remains from the first two phases of occupation of the site (Fig. 4, data from systematically collected samples, see Xue et al. 2022; Dal Martello 2020; Xue 2010). This would suggest that it was actively exploited (Dal Martello 2020). Other important finds include high quantities of charred hemp seeds (*Cannabis* sp.), *Prunus* pit fragments, soybean (*Glycine max*), and a few nutlets of buckwheat, *Vitis* and one *Cucumis melo* seed (Table 1; Xue 2010; Dal Martello 2020). Zooarchaeological analyses at the site showed that animal husbandry, especially pig but also sheep, goat and possible *Bos gaurus*, was practiced at Haimenkou, and fishing and hunting also contributed to the diet (Wang 2018; Table S2). The hypothesis of gaur management raises interesting questions because today its domesticated form, the gayal (*Bos frontalis*), is found amongst minority peoples from Northeast India and Myanmar (Simoons and Simoons 1968; Shaller 1967; Larson and Fuller 2014); Murphy and Fuller 2018: Fig. 8). If the Haimenkou finds represent initial gayal herding, then this was earlier than usually assumed (e.g. Larson and Fuller 2014) and might have spread from there southward and westward to Myanmar and India.

Only one context was floated at Shifodong (Zhao Zhijun, personal comment 2018), and this contained rice, rice husks, millet remains, and two types of unidentified legumes and a couple of *Chenopodium* sp. seeds (Zhao 2010). Upon further examination of the extracted materials by the author, one of the legumes has been identified as *Tamarindus* cf *indica* (Dal Martello 2020), an arboreal Fabaceae that is typically regarded as native to the African continent and only later reaching China through dispersal through India. However, recent finds of archaeological wood remains and linguistics analyses from India have raised the possibility that a native population existed in the region (Asouti and Fuller 2008:104; Fuller 2007). The finds from Shifodong would further support this hypothesis.

Finally, a thick pile of rice grains (reportedly 7 kg corresponding to over 800,000 grains) mostly found inside a charred bamboo basket in the corner of a house, were found in 1990 during the excavation of the site of Yingpanshan, in Changning county, Western Yunnan (Xiang et al. 2015).

#### First millennium B.C.

A comparatively richer dataset of archaeobotanical results is available from sites in Yunnan dating to the first millennium B.C., and especially those associated with the Dian Kingdom in central Yunnan, where both systematic flotation during excavation and limited flotation during surveys has been applied (Table 1; Figs. 3 and 4; Dal Martello et al. 2021). The archaeobotanical assemblages from these sites are characterised by the presence of a high diversity of species, including several domesticated cereal crops (mostly rice, foxtail millet, and wheat) and other economic species, including soybean, *Prunus* fruits and nuts. These other economic species never account for more than 5% of the assemblage (Dal Martello 2020; Dal Martello et al. 2021). Additionally, *Chenopodium* sp. has been reported in high quantities at Guangfentou, however, its status as a weed or a crop at this site has not been investigated (Li and Liu 2016) and 149 grains of buckwheat have been reported from Xueshan (Wang 2014). Presumed buckwheat grains have also been reported from a site in the upper Lancang basin, in Northwest Yunnan, Zongzan, dated through cultural association to c. 1000- 700 B.C. (Chen et al. 2019; Li 2016); however, no photos of these presumed buckwheat grains from Zongzan nor any other information on the archaeobotanical assemblage have been published so far.

Archaeobotanical remains from the third period of occupation at the site of Haimenkou, in northwest Yunnan, show a continuation of the mixed-crop assemblage found in the previous two periods of occupation. This assemblage illustrates a decrease in abundance of rice (which, although is found in high absolute counts from one context only, decreases stratigraphically and it was not reported from samples from the uppermost cultural layer) and millet remains through time in favour of wheat, and a high presence of *Chenopodium* sp. (Fig. 4; Xue et al. 2022; Xue 2010; Jin 2013; Dal Martello 2020).

Finally, a site on the western edge of the province, on the Lancang River, Shilingang, has undergone both archaeobotanical and isotopic analyses (Li et al. 2016; Zhang et al. 2017; Ren et al. 2017). Although the archaeobotanical remains recovered were not numerous, grains of rice and millets were reported. Isotopic analyses on human bones from the site also provided both C3 and C4 signatures, indicating a mixed subsistence strategy and possibly reflecting the mixed rice-millets systems detected from ancient macro-botanical remains from previous and contemporaneous sites in Yunnan (Zhang et al. 2017). Additionally, Zhang and colleagues (2017) reported the presence of microbotanical remains of tubers, roots, palms, and acorns. They proposed that people at Shilingang practiced a broad-spectrum subsistence strategy, taking advantage of the varied and abundant local resources. Isotopic analyses on human bones from two other cemeteries, Jinlianshan, in the Dian Basin (Zhang 2011), and Mayutian in southern Yunnan (Zhang et al. 2014), both dated to the first millennium B.C., also show a mix of C3 and C4 signatures, with a prevalence for C3 for Jinlianshan (Tables 1 and S2).

## Tracing the development of agricultural practices in Yunnan: A new synthesis

**Fig. 5:**
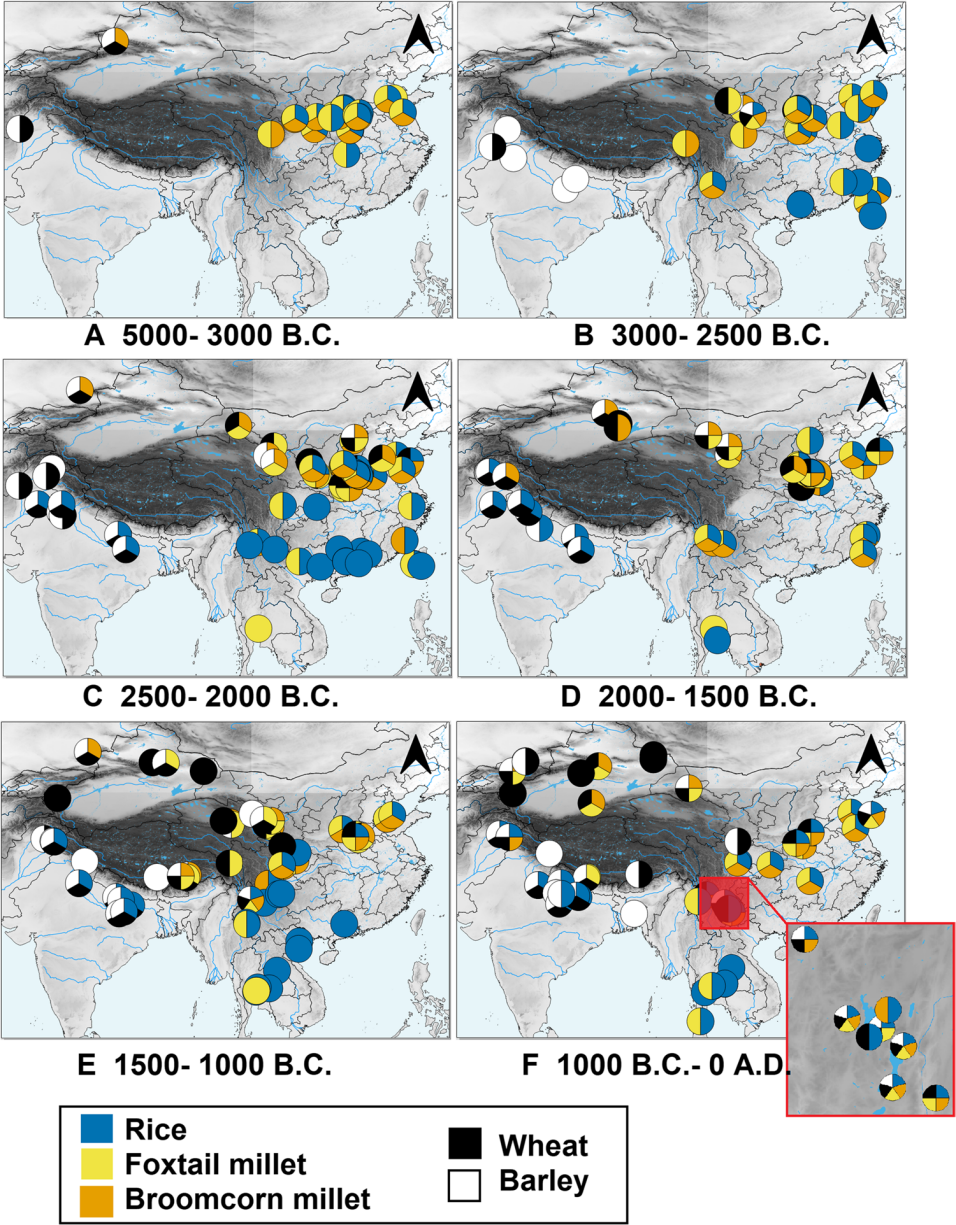
Map showing presence/absence data of main cultivated crops (rice, foxtail and broomcorn millets, wheat and barley) in relation to the spread of agriculture to and from Yunnan province. Data from Fuller et al. (unpublished). Sites are plotted according to the median date within each site chronological range. Made with QGIS 3.16.1

According to the available evidence, settled agricultural villages were present in Yunnan as early as the mid-third millennium B.C. (Table 1). The economy practiced at these sites was based on the mixed cultivation of dry and wetland cereals, mostly foxtail millet and rice, the use of the nearby water reservoirs for fishing as well as possibly pig rearing, and the gathering of local wild resources. Local fruit, nuts, and pulses, despite being recovered in much lower quantities than cultivated crops, are found ubiquitously throughout different periods of occupation and areas, suggesting their collection was an important component of the overall subsistence strategy. A similar type of mixed, millet-rice agricultural system has been reported from earlier sites in central China, including Nanjiaokou, Baligang, and Chengtoushan (Stevens and Fuller 2017; Weisskopf 2014; Nasu et al. 2012; Fig. 5), from where it could have spread to Yunnan through Sichuan (D’Alpoim Guedes 2013). This type of mixed crop system is well suited to the vertical zonation of Yunnan, with each valley surrounded by hills and mountains and thus having lowlands and highlands co-occurring at relatively close distances. This allows for a variety of environmental niches to co-exist close to each other, providing suitable growing conditions for crops requiring different ecological regimes. The ceramic assemblages retrieved at sites dating to the third millennium B.C. shows a high percentage of vessels apt to cook liquid substances, suggesting boiling was an important part of the local cooking tradition. This is in line with the division between a boiling and steaming tradition, characteristic of East Asia, distinct from the nearby baking and roasting tradition of South Asia (Fuller and Rowlands 2009, 2011).

### The introduction of wheat and barley

In the mid-second millennium B.C., wheat and barley are introduced to Yunnan, as attested by the presence of grains of both crops at the sites of Haimenkou. Wheat grains at Haimenkou have been directly dated to 1440–1310 cal B.C., corresponding to the second period of occupation of the site (Xue 2010; Li and Min 2014). Here, in conjunction with the appearance of wheat and barley, there is an increase in the number of dwellings and tools interpreted for cutting down trees and processing wood (possibly used to build the pile dwellings) and increased remains of sheep/goat animal bones (Wang 2018). This suggests an increased population and links with agropastoralists from western China, which were possibly responsible for the introduction of the two crops, either through direct migration into Yunnan or through contact and trade. However, it does appear that sheep/goat arrived before wheat, as it was present at Haimenkou since the initial occupation, as well as at the earlier nearby site of Baiyangcun (YPM 1981), dated to 2650- 1690 cal B.C. (Dal Martello et al. 2018).

There is still no consensus on the route or routes or even on whether the two crops dispersed to China together, with implication on how they then reached Yunnan. Especially for barley, both a northern route together with wheat, through the Inner Asian Corridor and Northwest China (i.e. Frachetti 2013; Spengler et al. 2014; Stevens et al. 2016; Kuzmina 2008), and a southern route, separate from wheat, via South Asia through the Southern Himalaya and Tibetan Plateau (i.e. Liu et al. 2017, 2019; Lister et al. 2018), have been proposed.

Outside of China, along the proposed northern route, barley has been found at Ojakly in Turkmenistan at 1617–1498 cal B.C., Tasbas in Kazakhstan at 1437–1233 cal B.C., and Aigyrchal-2 in Kyrgyzstan at 1630–1497 cal B.C. (Spengler 2015). Recently, it has also been reported from Tongtian Cave, in the Altai Mountains, where naked barley seeds have been directly dated to c. 3200 B.C., and wheat seeds to c. 3000 B.C. (Zhou et al. 2020), significantly predating the arrival of the crops in the region. The discovery of wheat and barley at Tongtian Cave is significant in trying to reconstruct the spread of the crops to China, as previously interior finds on the Northeastern Tibetan Plateau, dated to earlier than finds in Xinjiang: at Xiasunzhai, on the Northeastern Tibetan Plateau, barley grains furnished a date of 2136–1959 cal B.C. (Liu et al., 2017); in Xinjiang, barley has been reported from Sidaogou and Yanghai, dating 978–831 cal B.C. and 750–405 cal B.C. respectively (Liu et al. 2017).

The hypothesis for a separate, southern route of dispersal for barley is based on genetic analysis of modern barley landraces reconstructing a dispersal of barley from South Asia via the Himalaya into Southwest China, as well as an early date for barley from Kanispur, in Kashmir, 2467–2236 cal B.C. (Lister et al. 2018; Liu et al. 2017; Pokharia et al. 2018). Barley grains have also been reported at Chirand in Bihar, in the Lower Ganges valley, dated to 1920–1660 B.C. (Mittre 1972; Fuller 2011), and in Nepal, at the Chokhopani site, from ca. 1000 B.C. (Knörzer 2000). At Khog gzung and Bangtangbu, on the Southwestern Tibetan Plateau, barley dated to 1393–1211 cal B.C. and 1263–1056 cal B.C. respectively (Liu et al 2017; Lister et al 2018). Within Southwest China, further evidence for wheat and barley has been found at the sites of Qugong and Changguogou, on the Central Tibetan Plateau, dating to c. 1600–1400 B.C. (Gao et al. 2021; Fu 2001; Liu et al. 2016); at A’shaonao, in northern Sichuan (c. 1400 B.C.; d’Alpoim Guedes et al. 2015); and wheat only has been reported from Karuo, on the Northeastern Tibetan Plateau (c. 1600 B.C.; Liu et al., 2016).

At present, except for Shangxihe where wheat has been directly dated to 1095–933 cal B.C. at the earliest occurrence and was not associated with barley grains (Yao et al. 2020), at all other early sites in Yunnan barley is found in association with wheat grains. At these sites, barley is present in negligible quantities in comparison to wheat, possibly indicating it was grown secondarily to wheat.

Finally, while both naked and hulled barley varieties have been reported from Northern India, Pakistan and Central Asia, all early reports of barley from China are of the naked variety (Liu et al. 2017). The recent analyses of barley grains from Dian sites individuated the presence of hulled barley at Dayingzhuang (Dal Martello et al. 2021); this could imply a secondary, later spread of hulled, six-row barley, possibly through a southern route.

The current scattered data and the many geographical gaps (Fig. 5) present both from Northwest China, and the Southern Himalayan-northeast India regions do not allow us to fully resolve the issue of the route(s) through which the crops dispersed to China and how they reached Yunnan; the subordinate relationship of barley to wheat in Yunnan archaeobotanical assemblages suggest that whether or not the two crops dispersed to China separately, they then came together outside of Yunnan and reached the province as a package, either through migration or trade, which could, in turn, have come from several directions. The limited archaeobotanical data from second millennium B.C. Yunnan hinders the clarification of this issue, and thus only future, more systematic work will clarify the exact routes and way for the introduction of these two crops not only to Yunnan but also to China as a whole.

### Local resources

#### Buckwheat

Reports of ancient buckwheat are generally rare within the archaeological record, but thanks to the increasing deployment of flotation in recent years, finds of ancient macro-botanical remains of buckwheat, either common buckwheat (*Fagopyrum esculentum*) or bitter buckwheat (*Fagopyrum tataricum*), have started to accumulate, and come mostly from the broader Southwest China region. In Yunnan, buckwheat grains have been reported from Haimenkou (6 grains of *Fagopyrum* cf *esculentum*; 1600–400 B.C., Xue 2010; Dal Martello 2020); Xueshan (149 grains of *Fagopyrum esculentum*, c. 700 B.C.- 100 A.D.; Wang 2014), and possibly Zongzan (unclear number of grains and referred to only as buckwheat; Li 2016). Outside of Yunnan province, buckwheat grains have been reported from Qugong (1 grain of *Fagopyrum tataricum;* 1750–1500 cal B.C.; Gao et al. 2021); Donghuishan (3 grains identified only as “buckwheat”; the only instance of directly dated remains, 1610–1450 cal B.C.; Wei 2019); Yingpandi (1 grain of *Fagopyrum* sp.; c. 1550 B.C.; Jia 2012); Mebrak/Phudzeling (19 grains of *Fagopyrum tataricum* and 23 grains of *Fagopyrum esculentum*; c. 1000 B.C.- 400 A.D.; Knörzer 2000); Bangga (13 grains of *Fagopyrum* sp.; c. 133 A.D.; Lu et al. 2020), and Kaerdong (2 grains of *Fagopyrum tataricum*; c. 455- 700 A.D.; Song et al. 2018).

Although the origin of domesticated buckwheat is still not well understood, the region of Southwest China has been proposed as one possible centre of domestication for both common buckwheat (*Fagopyrum esculentum*) and bitter buckwheat *(Fagopyrum tataricum*; i.e. Ohnishi and Konishi 2001; Konishi et al. 2005; Ohnishi 1991; Ohnishi 1998; Ohnishi 2004; Ohnishi and Yasui 1998; Ohnishi and Tomiyoshi 2005; Ohnishi and Matsuoka 1996; Konishi and Ohnishi 2007; Boivin et al. 2012; Weisskopf and Fuller 2013). Modern wild populations of *F. esculentum* ssp. *ancestralis* are highly restricted to mid-elevations (1000–1500 m asl) on rocky slopes about the Jinsha River (Yunnan) and Yalong River (Sichuan), i.e. (Ohnishi, 1991; 1998; 2004; Ohnishi and Yasui 1998; Ohnishi and Konishi 2001; Ohnishi and Tomiyoshi 2005; Konishi et al. 2005). By contrast the wild *F. tartaricum* ssp. *potanini* is much more widespread around the southern and southwestern Tibetan plateau. Historical linguistic inferences from East Bodish languages (in Bhutan) suggest that the Eastern Himalayas region is a possible center of domestication of bitter buckwheat (*Fagopyrum tartaricum*) more than 2500 years ago, with *F. esculentum* adopted secondarily (Hyslop and d’Alpoim-Guedes 2020). Recent ancient macro-botanical remains of buckwheat (*F. esculentum*) from the second millennium B.C., as noted above, give additional support to a southwestern Chinese origin.

An alternative view has proposed North China as a possible centre for the domestication of buckwheat from the fifth millennium B.C. (Hunt et al. 2018). This earlier, northern hypothesis is based on a review of finds of pollen, and some starch grains, from lake sediments, paleo-soils, and archaeological sites. Pollen and starch grains, however, lack the possibility of being directly dated to prove their antiquity, contrary to macro-botanical remains. Several wild *Fagopyrum* species (between 15 and 20) are distributed broadly across southern-central China and Tibet (Campbell 1997; de Klerk et al. 2015). Although identification *Fagopyrum* pollen seems reliable, caution may be warranted over species-level identification of *F. esculentum* (see de Klerk et al. 2015)*.* Additionally, pollen and starch grains not recovered from archaeological contexts do not necessarily represent direct human activities, and could simply indicate the presence of wild *Fagopyrum* population nearby. At the sites across the Central and Southern Tibetan Plateau, as well as at Haimenkou and Xueshan, buckwheat grains were found together with other cultivated cereals, suggesting they might have been exploited for food. However, the lack of widespread publication of photos and measurements of archaeological buckwheat grains, makes it difficult to compare and evaluate them in the context of its possible domestication; more archaeobotanical work is needed to understand past use and the domestication trajectory of this species.

#### Fat hen (*Chenopodium album*)

*Chenopodium album*, commonly known as fat hen, is cultivated today as a minor winter crop in the southern Himalayas, especially important as insurance against famine (Partap and Kapoor 1985a, 1985b, 1987) and on parts of the Tibetan Plateau (Kang et al. 2013, 2014), as well as in highland areas of Taiwan, among the Formosan tribes, for both its grains and leaves (Fogg 1983). The origin of this species is not well understood, and although *Chenopodium* grains are reported in many archaeobotanical reports from archaeological sites in China, these usually account for a very small part of the overall assemblage and are thus categorised as a dryland weed. At Haimenkou, *Chenopodium* is not only found in high quantities, but its strong association with other cultivated cereals, rice and foxtail millet, indicates that the species was actively exploited and considered a food resource (Dal Martello 2020). *Chenopodium* remains retrieved from other sites in Southwest China have also prompted scholars to hypothesize it was actively collected and/or cultivated and exploited as a food resource, including at Guiyuanqiao, in Sichuan, dated to c. 3100–2600 B.C. d'Alpoim Guedes and Wan 2015; Gao 2021). Large quantities of *Chenopodium* grains have also been reported from sites in India dating to the Harappan Rojdi period (c. 2500–1700 B.C.; Weber 1989). The lack of systematic morphological, morphometric, and genetic analyses of archaeobotanically preserved *Chenopodium* grains from archaeological sites in Eurasia hinders our understanding of this species domestication trajectories as well as its possible role in the overall agricultural production at the sites where it was retrieved.

### Seasonal intensification of agricultural production during the Dian

Archaeobotanical remains from sites linked with the Dian Kingdom in central Yunnan show a distinct, multi-cropping system based on the cultivation of the summer crops of rice in the lowlands, grown in a semiaquatic regime, and foxtail millet, possibly alternating with wheat, in the surrounding hills (Dal Martello et al. 2021). Historical texts point to the presence of both summer and winter wheat and barley varieties in Central China from at least the first millennium B.C. (Liu et al. 2017). During the first millennium B.C., the climate in Yunnan was similar to the current conditions, and the biogeographical vertical zonation would have offered optimal growing conditions for this type of diversified two-season agriculture, based on the cultivation of lowland rice, and millet-winter wheat in the surrounding hills, that still characterizes the province today (Zhao 1986; Dal Martello et al. 2021). The rich water availability from lakes and rivers and the mild climate in the lowlands would also have allowed for continued rice production, but despite historical accounts describing irrigation practices in central Yunnan from the first century A.D., the present archaeobotanical data have not conclusively shown when these practices began and how and when they intensified through time (Dal Martello et al. 2021).

The continued presence of millet and rice after the introduction of wheat and barley shows that they did not replace existing crop systems, but instead, were incorporated into them, allowing for agricultural diversification. Climatic instability might have played a role in pushing for the establishment of a diversified agricultural system. In addition to cultivated cereal crops, *Chenopodium* (which could be grown either as a summer or a winter crop), and local resources (legumes, wild fruits and nuts) also continue to be part of the archaeobotanical assemblages, further suggesting that the most successful food production strategy in the area was to exploit the widest range of resources available, including both introduced crops and local resources.

## Yunnan early agricultural systems and the spread of agricultural crops to mainland Southeast Asia

Systematic archaeobotanical analyses at early sites in Yunnan, such as Baiyangcun, Dadunzi, and Haimenkou, have shown that here, third and second millennium B.C. agricultural production was based on the mixed cultivation of rice and millet; additionally, rice was grown in a wetland regime, as attested by the recovery of typical wetland weedy taxa, such as *Fimbristylis* sp., and *Scirpus* sp. associated with rice remains.

To date, the earliest attested agricultural systems in mainland Southeast Asia, instead, were based on rainfed cultivation of foxtail millet only, as reported from Non Pa Wai in central Thailand around ca. 2400 B.C., as well as at Non Mak La (ca. 2100–1450 B.C.) and Nil Kham Haeng (ca. 1350–800 B.C.; Weber et al. 2010). Rice is reported from archaeological sites later than the first appearance of millet: at Khok Phanom Di at ca. 2000–1400 B.C. (Higham and Thosarat 2012), at Non Mak La at ca. 1450–700 B.C., Non Pa Wai at ca. 1000–700 B.C. and Nil Kham Haeng at ca. 800–500 B.C. Here it has been proposed that it was possibly traded (Pigott et al. 2006: 166) or cultivated in a regime resembling that of foxtail millet (Castillo 2017:344). Other sites dating to the first millennium B.C. where rice grains have been retrieved include Ban Non Wat, Khao Sam Kaeo and Phu Kao Thong. At these sites, field weeds typical of dryland cultivation (i.e. *Acmella paniculate*) were associated with the rice grains, suggesting that in this area the crop was initially cultivated in a dryland regime (Higham 2014; Wohlfarth et al. 2016; Castillo et al. 2016; Castillo 2017, 2018), in constrast to the attested wetland rice cultivation from Yunnan. The earliest occurrence of both foxtail millet and rice is found at Rach Nui (1845–1385 B.C.) on the southern coast of Vietnam, where they were not cultivated but possibly traded from nearby (Castillo et al. 2018; Oxenham et al. 2015).

Further supporting an initial dryland agricultural system for mainland Southeast Asia, recent Austroasiatic linguistic reconstruction showed a closer relationship with terms describing dryland/hilly cultivation, and riverine resources, rather than lowland (irrigated) rice cultivation (i.e. Blench 2005; Sidwell and Blench 2011; Fuller 2011). The “Southeastern Riverine Hypothesis” proposed that Austroasiatic speakers’ homeland was in the middle Mekong (Lancang) Basin, and their subsistence relied on tubers, such as *Colocasia esculenta*, and riverine resources exploitation, with rice being adopted after the language family had already started to diversify (Sidwell and Blench 2011).

The contrast in cultivation ecology, a wetland regime in Yunnan, as opposed to a dryland system in mainland Southeast Asia, attested both by the recent archaeobotanical and linguistic data, suggest that Austroasiatic speakers might not have been the main source for crop dispersal to the region, as previously hypothesised. An alternative route for cereal crops dispersal from China to mainland Southeast Asia could be the southern China coast, i.e. Fujian and Guangdong (e.g. Castillo 2017; Castillo and Fuller 2010; Fuller et al. 2010; Qin and Fuller 2019). Remains of rice and millets have been reported from the sites of Gantouyan (ca. 3500–1000 B.C.; Lu 2009), Huangguashan, Pingfengshan, and Nanshan in Fujian, and rice remains were reported also from Shixia and Layuan, all dating from the third millennium B.C. (Yang et al. 2018, 2017; Deng et al. 2018). The hilly landscape of this area might have facilitated the development of upland rainfed rice varieties (Deng et al. 2018), and although it has been proposed that rice at Shixia and Layuan, was cultivated in a dryland regime (Yang et al., 2018), however, no associated archaeobotanical remains have been found to support this hypothesis. Thus, the question of when and where dryland rice cultivation developed and how it spread to mainland Southeast Asia needs further evidence to be conclusively determined.

In addition to cereal crops, bronze technology diffused from China to Southeast Asia, either through Yunnan (e.g. White and Hamilton 2009), or Central China (e.g. Pryce 2017; Pigott and Ciarla 2007; Higham, 1996), or possibly through both routes (Ciarla 2013) later than the initial spread of agricultural crops. This shows that agricultural and technological innovations in the region emerged as a result of multi-directional and extended through time contacts and movements between China and mainland Southeast Asia, rather than a single and defined southward dispersal. The present scarcity of systematic archaeobotanical data in archaeological research and the many geographical and chronological gaps still present from both Yunnan-broader South China, and mainland Southeast Asia do not allow us to conclusively solve this issue, and further data is needed to clarify the route(s) and timing through which cereal crops reached mainland Southeast Asia.

## Conclusion

Given the growing number of systematic archaeobotanical analyses and radiocarbon dating from prehistoric sites across Yunnan, it is now possible to evaluate previous theories on agricultural dispersal and development. The picture that has started to emerge shows a complex developmental trajectory that extended through multi-directional connections and brought technological and cultural innovation to the region. Yunnan was an important area for early agricultural dispersal of both rice and the millets, which have been found together since the earliest attested agricultural villages, dating to the mid-third millennium B.C. The established mixed-crop agricultural system continues to be practiced through the successive millennia, and after the introduction of wheat and barley in the mid-second millennium B.C., this system expanded to incorporate newly arrived crops. This possibly allowed for a seasonal intensification of the agricultural production during the Dian based on the cultivation of rice in the lowlands, and millet and wheat in the surrounding hills, in the first millennium B.C.

By comparing archaeobotanical evidence from Yunnan and mainland Southeast Asia, previous theories of a single southward dispersal of cultivated rice linked with Austroasiatic speakers originating in Yunnan and bringing agriculture into mainland Southeast Asia find little support. The recent accumulation of archaeological, archaeobotanical, and linguistic data, instead, point to a complex series of overlays across several millennia, with multiple source for the southward spread of cereal crops from China into mainland Southeast Asia. The many geographical gaps in our data and the lack of widespread reliable radiocarbon and systematic archaeobotanical investigation from both south China and mainland Southeast Asia do not allow to conclusively establish the development history of differing rice cultivation ecology and the precise routes through which this innovation dispersed, neither when intensive rice irrigation started in Yunnan and future research should address these gaps.

## Supplementary Information

Below is the link to the electronic supplementary material.

Supplementary file1 (PDF 523 kb)

Supplementary file2 (PDF 515 kb)

## Data Availability

Data summarised and presented in this paper is available as supplementary material.
