# Supplementary material for: The origins of multi-cropping agriculture in Southwestern China: Archaeobotanical insights from third to first millennium B.C. Yunnan
Source: Asian Archaeol. 2022 May 25;6(1):65–85. doi: 10.1007/s41826-022-00052-2 (PMC9373101; doi:10.1007/s41826-022-00052-2)
Supplement: Supplementary file 1 — Supplementary file1 (PDF 523 kb) [file 41826_2022_52_MOESM1_ESM.pdf]

Table S1. Summary of reported radiocarbon dates from sites in Yunnan with evidence for ancient plant remains.

Site	Lab No.	Original Samples No.	Sample Material	Archaeological context	h.l. 5730	h.l. 5568	68.20% cal BC	95.40% cal BC	Method	REFERENCES
Baiyangcun	ZK-0220	n/a	wood charcoal	F3 posthole2	3770±85	3663±85	2200-1920	2300-1770	LSC	YPM 1981
	ZK-0330	n/a	wood charcoal	Unno. posthole in trench 7	3675±85	3571±85	2030-1770	2190-1690	LSC	
	Beta-501547	2013YBB (5)	Rice grain	Layer 5		3480±30	1880-1750	1890-1690	AMS	Dal Martello et al. 2018
	OxA-33286	2013YBBT2(8)c S4	Rice grain	Layer 8		3743±29	2210-2060	2280-2030	AMS	Dal Martello 2020
	OxA-33290	2013YBBT2(8)c S4	Rice grain	Layer 8		3764±28	2280-2130	2290-2040	AMS	
	OxA-33291	2013YBBT2(9)c S3	Rice grain	Layer 9		3718±29	2200-2040	2210-2030	AMS	
	OxA-33327	2013YBBT2(9)c S3	Rice grain	Layer 9		3689±35	2140-2030	2200-1960	AMS	
	OxA-33328	2013H118	Rice grain	H118 （sealed by layer 15）		3731±30	2200-2040	2270-2030	AMS	
	OxA-33293	2013H118	Rice grain	H118 （sealed by layer 15）		3735±29	2200-2050	2270-2030	AMS	
	OxA-33287	2013YBBT2(17) S4	Rice grain	Layer 17		3916±29	2470-2340	2480-2290	AMS	
	OxA-33292	2013YBBT2(17) S3	Rice grain	Layer 17		3898±29	2470-2340	2470-2290	AMS	
	SUERC-73806	2013YBBT2(20)	Millet grain	Layer 20		3929±23	2480-2340	2490-2330	AMS	
	OxA-33288	2013YBBT2(21)c S4	Rice grain	Layer 21		3958±30	2570-2410	2570-2340	AMS	
	OxA-33289	2013YBBT2(21)c S4	Rice grain	Layer 21		4035±28	2580-2490	2630-2470	AMS	
	SUERC- 73802	2013YBBT2(24) S3	Millet grain	Layer 24		4110±34	2860-2580	2879-2570	AMS	
Haidong	BK89079?	n/a	n/a	n/a	4235±150	4115±150	2890-2490	3090-2200	LSC?	He 1990<br>Xiao 2001<br>Yao 2010
Xinguang	BK94072	T1104F5	peat	F5	4030±80	3658±70	2140-1940	2280-1780	LSC	YPICRA 2002
	BK94073	T1104I2	peat	I2	3830±70	3668±70	2140-1940	2290-1880	LSC	Li et al. 2016
	BK94074	T1105(8)	peat	Layer 8	3775±70	3721±70	2280-1980	2350-1920	LSC	
	BK94075	T1105(6)	peat	Layer 6	3765±70	3916±80	2560-2280	2620-2140	LSC	
Dadunzi	ZK-0229	n/a	wood charcoal	F5 posthole 12	3210±90	3119±90			LSC?	YPM 1977

Site	Lab No.	Original Samples No.	Sample Material	Archaeological context	h.l. 5730	h.l. 5568	68.20% cal BC	95.40% cal BC	Method	REFERENCES
Dadunzi	n/a	2010MDT18-2-S1	Rice grain	Layer 2		3385±30	1740-1630	1750-1610	AMS	Jin et al. 2014
	n/a	2010MDT18-H3-2-S1	Rice grain	H3		3420±20	1750-1690	1870-1650	AMS	
	n/a	2010MDT18-4-S1	Rice grain	Layer 4		3540±35	1940-1780	1970-1750	AMS	
	n/a	2010MDT18-5-S1	Rice grain	Layer 5		3555±25	1950-1880	2010-1770	AMS	
	n/a	2010MDT18-7-S1	Rice grain	Layer 7		3555±25	1950-1880	2010-1770	AMS	
	n/a	2010MDT18-8-S1	Rice grain	Layer 8		3685±25	2140-2030	2190-1970	AMS	
	n/a	2010MDT18-9-S1	Foxtail millet grain	Layer 9		3665±40	2140-1970	2200-1920	AMS	
Haimenkou	ZK2335	CH T2 (4)	wood charcoal	Layer 4	2595±75	2520±75	800-540	810-420	LSC	CASS 1990
	not provided	T1005-4-s1	Rice grain	Layer 4		2400±20	490-400	540-400	AMS	Li and Min 2014
	not provided	T1003-4-s2	Wheat grain	Layer 4		2405±35	520-400	750-390	AMS	Min 2013
	not provided	T100454-s6	Foxtail millet grain	Layer 4		2435±03	730-410	760-400	AMS	
	not provided	T1003-5-s2	Wheat grain	Layer 5		2445±35	740-410	760-400	AMS	
	not provided	T1005-6-s4	Rice grain	Layer 6		2960±25	1220-1120	1270-1050	AMS	
	not provided	T1003-6-s2	Wheat grain	Layer 6		2975±45	1270-1120	1390-1040	AMS	
	not provided	T1003-6-s1	Wheat grain	Layer 6		3000±35	1290-1130	1390-1120	AMS	
	not provided	T1004-6-s3	Soybean	Layer 6		3045±40	1390-1230	1400-1220	AMS	
	not provided	T1004-6-s3	Foxtail millet grain	Layer 6		3050±30	1390-1260	1410-1120	AMS	
	not provided	T1003-7-s2	Wheat grain	Layer 7		3060±35	1400-1270	1420-1220	AMS	
	not provided	T1004-7-s6	Rice grain	Layer 7		3075±35	1400-1290	1430-1230	AMS	
	not provided	T1005-7-s2	Wheat grain	Layer 7		3095±30	1420-1300	1430-1270	AMS	
	not provided	T1005-7-s1	Wheat grain	Layer 7		3125±30	1440-1310	1500-1290	AMS	
	not provided	T1004-7-s3	Foxtail millet grain	Layer 7		3210±30	1510-1440	1600-1410	AMS	
	not provided	T1003-7-s2	Rice grain	Layer 7		3240±40	1610-1450	1620-1430	AMS	
	not provided	T1005-8-s2	Wheat grain	Layer 8		3105±25	1420-1300	1440-1290	AMS	

Site	Lab No.	Original Samples No.	Sample Material	Archaeological context	h.l. 5730	h.l. 5568	68.20% cal BC	95.40% cal BC	Method	REFERENCES
Haimenkou	not provided	T1005-8-s2	Rice grain	Layer 8		3250±35	1610-1460	1620-1440	AMS	
	not provided	T1003-8-s2	Foxtail millet grain	Layer 8		3275±35	1610-1460	1620-1450	AMS	
	not provided	T1003-9-s2	Foxtail millet grain	Layer 9		3230±40	1600-1440	1620-1420	AMS	
	not provided	T1003-9-s2	Rice grain	Layer 9		3275±35	1610-1500	1640-1450	AMS	
	not provided	T1003-10-s1	Rice grain	Layer 10		3380±25	1730-1630	1750-1620	AMS	
	BA081094	2008JHAT1304(5)	seed	Layer 5		3000±35	1290-1130	1390-1120	AMS	
	BA081095	2008JHAT2121(5)	rhizome	Layer 5		2200±35	360-200	380-170	AMS	
	BA081096	2008JHAT2002(6)	charred wheat	Layer 6		2435±35	730-410	760-400	AMS	
	BA081097	2008JHDT1005(6)	charred grain	Layer 6		3020±35	1380-1210	1400-1120	AMS	
	BA081098	2008JHDT1304(6)	vegetable fiber	Layer 6		3075± 35	1400-1290	1430-1230	AMS	
	BA081099	2008JHAT2003(6)	charred rice	Layer 6		2930±35	1200-1050	1230-1010	AMS	
	BA081100	2008JHAT2003(6)	charred millet	Layer 6		2940±35	1220-1080	1260-1020	AMS	
	BA081101	2008JHDT2003(7)	charred millet	Layer 7		3550±40	1950-1780	2020-1750	AMS	
	BA081102	2008JHAT2505(7)	rhizome	Layer 7		3205±35	1510-1430	1610-1410	AMS	
	BA081103	2008JHDT1205(8)	charcoal	Layer 8		3605±40	2030-1560	2130-1830	AMS	
	BA081104	2008JHDT1005(9)	charcoal	Layer 9		3345±35	1690-1560	1740-1530	AMS	
	BA081105	2008JHDT1004(9)	wood charcoal	Layer 9		4210±35	2900-2700	1740-1530	AMS	
	BA081106	2008JHDT1003(10)	rhizome	Layer 10		4485±35	3340-3090	3350-3030	AMS	
Shifodong	ZK-3198	2003GST13(4)D	wood charcoal	Layer 4D		2977±59	1290-1110	1400-1020	C14	CASS 2005
	ZK-3199	2003GST13(4)D	wood charcoal	Layer 4D		2998±47	1370-1120	1400-1050	C14	Liu and Dai 2008
Shangxihe	Beta488056	n/a	[i]	4		2200 ± 30		391 - 275	*AMS?**	Yao et al. 2020
	Beta488057	n/a	[i]	5		2470 ± 30		591 - 416	AMS?	
	Beta472799	n/a	[i]	5A		2530 ± 30		678 - 542	AMS?	
	Beta472800	n/a	[i]	5A		2580 ± 30		677 - 551	AMS?	

Site	Lab No.	Original Samples No.	Sample Material	Archaeological context	h.l. 5730	h.l. 5568	68.20% cal BC	95.40% cal BC	Method	REFERENCES
Shangxihe	Beta472801	n/a	[i]	6		2500 ± 30		766 - 604	AMS?	
	Beta472802	n/a	[i]	6		2490 ± 30		764 - 605	AMS?	
	Beta472803	n/a	[i]	6		2530 ± 30		776 - 595	AMS?	
	Beta472805	n/a	[i]	7		2520 ± 30		789 - 668	AMS?	
	Beta472807	n/a	[i]	8		2540 ± 30		801 - 752	AMS?	
	Beta472808	n/a	[i]	8		2590 ± 30		812 - 767	AMS?	
	Beta472810	n/a	[i]	9		2780 ± 30		976 - 840	AMS?	
	Beta472809	n/a	[i]	10A		2540 ± 30		804 - 778	AMS?	
	Beta472811	n/a	[i]	10A-1		2930 ± 30		1092 - 1019	AMS?	
	Beta472812	n/a	[i]	10A-1		2960 ± 30		1094 - 1024	AMS?	
	Beta472813	n/a	[i]	10A-2		2850 ± 30		1052 - 932	AMS?	
	Beta472816	n/a	[i]	10A-2		2840 ± 30		1050 - 926	AMS?	
	Beta472817	n/a	[i]	12		2920 ± 30		1116 - 1057	AMS?	
	Beta472818	n/a	[i]	12		2860 ± 30		1112 - 1060	AMS?	
	Beta472819*	n/a	Wood charcoal	13		3150 ± 30		1500 - 1311	AMS?	
	Beta472820	n/a	[i]	13		2800 ± 30		1126 - 1071	AMS?	
Shizhaishan	BA 091158	n/a	seed	Layer 6		n/a	n/a	779- 488	AMS?	Yao and Jiang 2012
Hebuosuo	Beta 312944	n/a	n/a	n/a		n/a	n/a	410-38	AMS?	Yao et al. 2015
	Beta 312945	n/a	n/a	n/a		n/a	n/a	200 BC- 40 AD	AMS?	Yao et al. 2020
	Beta 405371	n/a	n/a	n/a		n/a	n/a	734-400	AMS?	
	Beta 405368	n/a	[i]	Layer 3		2520 ± 30		674 - 539	AMS?	
	UCI 152059	n/a	[i]	Layer 3A		2565 ± 20		681 - 563	AMS?	
	Beta 405369	n/a	[i]	Layer 4		2510 ± 30		690 - 583	AMS?	
	Beta 405370	n/a	[i]	Layer 4A		2490 ± 30		716 - 602	AMS?	
	Beta 405371	n/a	[i]	Layer 5		2410 ± 30		750 - 645	AMS?	
	Beta 405360	n/a	[i]	Layer 5		2510 ± 30		781 - 627	AMS?	
	Beta 405361	n/a	[i]	Layer 6		2530 ± 30		798 - 741	AMS?	
	UCI 152061	n/a	[i]	Layer 6A		2870 ± 20		1104 - 945	AMS?	

Site	Lab No.	Original Samples No.	Sample Material	Archaeological context	h.l. 5730	h.l. 5568	68.20% cal BC	95.40% cal BC	Method	REFERENCES
Hebuosuo	Beta 405373	n/a	[i]	Layer 7		2920 ± 30		1186 - 1013	AMS?	
Anjiang	Beta 312943	n/a	n/a	Layer 6		n/a	n/a	770-650	AMS?	Yao et al. 2015
	Beta 312942	n/a	n/a	Layer 5		n/a	n/a	730-590	AMS?	
	BA 091156	n/a	n/a	Layer 3		n/a	n/a	640-430	C14?	
Shilinggang	LZU1468	n/a	Rice grain	Layer 4		2375±30	490-390	710-390	AMS	Li et al. 2016
	LZU1469	n/a	Rice grain	Layer 5		2480±30	760-540	780-430	AMS	
Dayingzhuang	Beta-501549	2017YHD 2	Wheat grain	Layer 2		100±30	modern	modern	AMS	Dal Martello 2020
	Beta-051550	2017YHD 4	Wheat grain	Layer 4		2380±30	485-400	727-393	AMS	Dal Martello et al. 2021
	Beta-051549	2017 YHD 5	Wheat grain	Layer 5		2430±30	726-414	750-405	AMS	

*Shangxihe Beta 472819 was excluded by the Yao et al. (2020) as it represents an old wood problem.

[i] According to Yao et al. 2020, some of the dates for Shangxihe and Hebosuo are based on the AMS dating of charred wheat, rice grains, and other charred seeds "recovered from either building postholes, floor surfaces or ash pits." Other dates derive from the radiocarbon dating of wood charcoal; however, no further indication is provided linking individual dates with the sample material.

References

CASS, Chinese Academy of Social Sciences, C14 Laboratory. 2005. Fangshexing tansu ceding niandai baogao (sai yi) 放射性碳素测定年代报告(三一) [Report on radiocarbon dating, vol. 31]. *Kaogu 考古 [Archaeology]* 7:57-61.

CASS, Chinese Academy of Social Sciences, Institute of Archaeology, Radiocarbon Laboratory. 1990. Fangshengxing tansu ceding niandai baogao (yiqi). *Archaeology* 7:663-668.

Dal Martello, Rita. 2020. Agricultural Trajectories in Yunnan, Southwest China: a comparative analysis of archaeobotanical remains from the Neolithic to the Bronze Age. UCL (University College London).

Dal Martello, Rita, Dorian Q Fuller, and Xiaorui Li. 2021. Two season agriculture and irrigated rice during the Dian: radiocarbon dates and archaeobotanical remains from Dayingzhuang, Yunnan, Southwest China *Archaeological and Anthropological Sciences* 13 (4):1-21.

Dal Martello, Rita, Rui Min, Chris Stevens, Charles Higham, Thomas Higham, Ling Qin, and Dorian Q Fuller. 2018. Early agriculture at the crossroads of China and Southeast Asia: archaeobotanical evidence and radiocarbon dates from Baiyangcun, Yunnan. *Journal of Archaeological Science: Reports* 20:711-721.

He, J. 1990. Tonghai Haidong cun beiqiu yizhi [The shell-midden site of Haidong in Tonghai]. In *Zhongguo Kaoguxue Nanjian (in Chinese)*, 304-305. Beijing: Cultural Relics Press.

Jin, Hetian, Xu Liu, Min Rui, Xiaorui Li, and Xiaohong Wu. 2014. Early subsistence practices at prehistoric Dadunzi in Yuanmou, Yunnan: new evidence for the origins of Early Agriculture in Southwest China. *The 'Crescent-Shaped Cultural-Communication Belt': Tong Enzheng's Model in Retrospect. Archaeopress, Oxford*.

Li, HaiMing, XinXin Zuo, LiHong Kang, LeLe Ren, FengWen Liu, HongGao Liu, NaiMeng Zhang, Rui Min, Xu Liu, and GuangHui Dong. 2016. Prehistoric agriculture development in the Yunnan-Guizhou Plateau, southwest China: Archaeobotanical evidence. *Science China Earth Sciences* 59 (8):1562-1573.

Li, K, and R Min. 2014. The site of Haimenkou: New research on the chronology of the Early Bronze Age in Yunnan. *The 'Crescent-Shaped Cultural-Communication Belt': Tong Enzheng's Model in Retrospect. An Examination of Methodological, Theoretical and Material Concerns of Long-Distance Interactions in East Asia. BAR International Series* 2679:123-132.

Liu, X, and Z Dai. 2008. 3000 Nian qian de Xueju Shenghuo: Gengma Shifodong Yizhi [Cave life from 3000 years ago: the site of Shifodong, Gengma]. *Zhongguo Wenhua Yichan* 6:84-87.

Min, Rui. 2013. Haimenkou yizhi zonghe yaniu [Comprehensive study of the Haimenkou site]. *Xueyuan* 15:6-9.

Xiao, MH. 2001. Yunnan Kaogu Shulu [Yunnan Archaeology]. *Kaogu [Archaeology] (in Chinese)* 12:1063-1075.

Yao, Alice. 2010. Recent developments in the archaeology of southwestern China. *Journal of Archaeological Research* 18 (3):203-239.

Yao, Alice, Valentín Darré, Jiang Zhilong, Wengcheong Lam, and Yang Wei. 2020. Bridging the time gap in the Bronze Age of Southeast Asia and Southwest China (long title). *Archaeological Research in Asia* 22:100189.

Yao, Alice, and Zhilong Jiang. 2012. Discovering the elusive Bronze Age settlements of the 'Dian'kingdom, China. *Antiquity* 86 (332):353-363.

Yao, Alice, Zhilong Jiang, Xuexiang Chen, and Yin Liang. 2015. Bronze age wetland/scapes: complex political formations in the humid subtropics of southwest China, 900–100 BC. *Journal of Anthropological Archaeology* 40:213-229.

YPICRA, Yunnan Provincial Institute of Cultural Relics and Archaeology. 2002. Yunnan Yongping Xinguang yizhi Fajue Baogao [Excavation Report of the site of Xinguang, Yongping, Yunnan]. *Kaogu Xuebao (in Chinese)* 2:203-204.

YPM, Yunnan Provincial Museum. 1977. Yuanmou Dadunzi Xinshiqi Shidai Yizhi [The Neolithic site of Dadunzi in Yuanmou]. *Kaogu Xuebao (in Chinese)* 1:43-71.

YPM, Yunnan Provincial Museum. 1981. Yunnan Binchuan Baiyangcun yizhi [The site of Baiyangcun in Binchuan, Yunnan]. *Kaogu Xuebao (in Chinese)* 3:349-368.
