# Supplementary material for: The origins of multi-cropping agriculture in Southwestern China: Archaeobotanical insights from third to first millennium B.C. Yunnan
Source: Asian Archaeol. 2022 May 25;6(1):65–85. doi: 10.1007/s41826-022-00052-2 (PMC9373101; doi:10.1007/s41826-022-00052-2)
Supplement: Supplementary file 2 — Supplementary file2 (PDF 515 kb) [file 41826_2022_52_MOESM2_ESM.pdf]

Table S2. Comparison of sites in Yunnan with evidence for ancient plant remains.

Latin names indicate systematic environmental sampling and analysis, common English names indicate the remains were hand-picked or systematic analysis is still to be undertaken.

Site	Location	Exc. Date	Exc. Area m^2	Estimated site size ha	Chronology	Features	House structure	Burial type	Ceramics	Main archaeobotanical remains	Zooarchaeological remains	References
**Baiyangcun** 白羊村	Middle Jinsha, Binchuan county	1973/74<br>2013/2014	290<br>100	10-20	AMS<br>2650-1690 cal BC	11 houses<br>14 hearts<br>48 pits<br>34 graves	wattle and daub	shaft pit: extended supine position	Baiyangcun type: Impressed-incised	*Oryza sativa*<br>*Setaria italica*<br>*P. miliaceum*<br>*Echinochloa* sp.<br>*Glycine soja*<br>*Vigna* sp.<br>*Cajanus* sp.<br>*Cucumis* sp.<br>*Euryale ferox*<br>*Vitis* sp.	Pig<br>Cattle<br>Goat/sheep<br>Wild boar<br>Black bear<br>Deer	YPM 1981;<br>Dal Martello et al. 2018;<br>Dal Martello 2020
**Haidong** 海东	Qilu Lake, Tonghai county	1988/89	372	3-8.5	c. 2500- 1750 BC	30 graves<br>40 hearths	shell mound site	shaft pit: supine position; extended facing East/ crouched facing South	Shizhaishan (Neolithic type): corded ware	Rice	Lacustrine resources; tortoise shells, and other unspecified animal bones	He 1990;<br>Xiao 2001;<br>Zhang and Hung 2010;<br>Yao 2010
**Xinguang** 新光	Upper Lancang, Yongping county	1993/94	1000	3-8	2500-1750 BC	21 pits<br>6 houses<br>1 moat<br>7 hearths	Semi-subterr./ wattle and daub		Xinguang type: Impressed-incised	Charred rice grains from G3	n/a	YPICRA 2002;<br>Yao 2010
**Dadunzi** 大墩子	Middle Jinsha, Yuanmou county	1972/73<br>1999- 2010	921	1.6	AMS<br>2200-1650 cal BC	15 houses<br>5 hearts<br>4 pits<br>37 graves	wattle and daub/ semi-subterr./ stilt houses	shaft pits/ stone cists/ urns	Dadunzi type: Impressed-incised	*Oryza sativa*<br>*Setaria italica P. miliaceum*<br>*Vigna* sp.<br>Cucurbitaceae	Pig<br>Dog<br>Cattle<br>Goat/sheep<br>Chicken<br>Muntjac<br>Deer<br>Lacustrine resources	Jin et al. 2014

Table S2. Comparison of sites in Yunnan with evidence for ancient plant remains.

Latin names indicate systematic environmental sampling and analysis, common English names indicate the remains were hand-picked or systematic analysis is still to be undertaken.

Site	Location	Exc. Date	Exc. Area m^2	Estimated site size ha	Chronology	Features	House structure	Burial type	Ceramics	Main archaeobotanical remains	Zooarchaeological remains	References
Xingyi 兴义	Qilu Lake, Tonghai county (Kunming)	2015/16	190	52	c. 2000-0 BC	47 floors<br>18 houses<br>24 graves<br>16 pits<br>4 streets<br>2 ditches<br>1 walled structure	n/a	Shaft pits/ urns: flexed	Shizhaishan (Neolithic type): corded ware	Acorns, *Oryza* sp. (unpubl.)	Abundant lacustrine resources: *Margarya* sp.	YPICRA 2017
Zongzan 宗咱	Langcan, Weixi county	2013	1600		c. 2000- 200 BC	Stone walls and structures				Buckwheat?	Cattle<br>Sheep<br>Deer<br>Pig<br>Monkey<br>Bear	Li 2016;<br>Chen et al. 2019
Yinpanshan 营盘山	Baoshan, Changning	1990	50	1	c. 1800 BC	n/a	Semi-subterr.			Rice		Xiang et al. 2015
Haimenkou 海门口	Middle Jinsha, Jianchuan county	1957<br>1978<br>2008	1350	10	AMS<br>1600-400 cal BC	Unknown no. of Houses<br>Pits<br>Hearths	wood pile-stilt houses		Early: Baiyangcun type: Incised-impressed<br><br>Late: Northwest China ceramics	*Oryza sativa*<br>*Setaria italica*<br>*P. miliaceum*<br>*Chenopodium* sp.<br>*Triticum aestivum*<br>*Hordeum vulgare*<br>*Fagopyrum* cf *esculentum*<br>*Cannabis* sp.<br>*Prunus* cf *persica*<br>*Prunus* cf *armeniaca*<br>*Quercus* sp.	*Sus domesticus*<br>*Ovis/Capra* sp.<br>*Canis familiaris*<br>*Bos gaurus*<br>*Cervus unicolor*<br>*Sus scrofa*<br>*Axis porcinus*<br>*M. muntjak*<br>*M. berezovskii,*<br>*Macaca* sp.<br>*Ursus* sp.<br>*Lepus* sp.<br>*Volpe* sp.	YPM 1958;<br>Xue 2010;<br>Jin 2013;<br>Li and Min 2014;<br>Wang 2018;<br>Dal Martello 2020
Mopandi 磨盘地	Middle Jinsha, Yongren county	1983	100	0.8	c. 1400 BC	2 houses<br>1 hearth<br>17 postholes<br>1 ditch<br>7 graves	wattle and daub, mostly rectangular, some circular	stone cists	Caiyuanzi type: Incised-impressed	Rice	Pig<br>Cattle<br>Goat/sheep<br>Dog<br>Chicken<br>Deer<br>Muntjac	YPICRA 2003;<br>Zhao 2003

Table S2. Comparison of sites in Yunnan with evidence for ancient plant remains.

Latin names indicate systematic environmental sampling and analysis, common English names indicate the remains were hand-picked or systematic analysis is still to be undertaken.

Site	Location	Exc. Date	Exc. Area m^2	Estimated site size ha	Chronology	Features	House structure	Burial type	Ceramics	Main archaeobotanical remains	Zooarchaeological remains	References
**Shifodong**<br>石佛洞	Middle Lancang, Gengma county	1982<br>2003	750	0.3	c. 1400-1100 BC	Several hearths	Cave site		Shifodong type: Incised-impressed	*Oryza sativa*<br>*Setaria italica*<br>*Chenopodium* sp.<br>*Tamarindus* cf *indica*<br>Indet. tree legume	Pig<br>Dog<br>Cattle<br>Deer<br>Horse?<br>Indet. birds/ fish species	Kan 1983;<br>Liu and Dai 2008;<br>Yao 2010;<br>Zhao 2010
**Nanbiqiao**<br>南碧桥	Lower Lancang	1982	n/a	0.3	c. 1250-970 BC		Cave site		Shifodong type	Rice	n/a	Kan 1983;<br>An 1999
**Shizhaishan**<br>石寨山	Dianchi Lake, Jinning county	1953<br>1955<br>1958<br>1960	204.3	0.05?	AMS<br>779-488<br>cal BC	28 graves		shaft pit: supine extended	Dian type: Incised-impressed	*Triticum aestivum*<br>*Oryza sativa*<br>*Setaria italica*	n/a	YPM 1963;<br>Yao and Jiang 2012
**Hebosuo**<br>河泊所	Dianchi Lake Jinning county	2014	12.5	31	AMS<br>1186-945 cal BC/<br>789-674 cal BC	n/a	Semi-subterr./<br>Pile-dwelling		Dian type	*Oryza sativa*<br>*Triticum aestivum*<br>*Setaria italica*<br>*P. miliaceum*<br>*Glycine max*	n/a	Yao et al. 2020;<br>Yang 2016;<br>YPICRA and Chicago 2019;<br>Yao et al. 2015;<br>Yao and Jiang 2012
**Shangxihe**<br>上西河	Dianchi Lake Jinning county	2014	500	n/a	AMS<br>1212-209 cal BC	n/a	Semi-subterr./<br>Pile-dwelling		Dian type	*Oryza sativa*<br>*Triticum aestivum*	n/a	Yao et al. 2020;<br>YPICRA and Chicago 2019
**Anjiang**	Dianchi Lake Jinning county	2008 2010/11	Survey	n/a	AMS<br>770- 430<br>cal BC	n/a			Shizhaishan/<br>Dian	*Oryza sativa*<br>*Triticum aestivum*<br>*Hordeum vulgare*<br>*Setaria italica*<br>*P. miliaceum*<br>*Chenopodium* sp.	n/a	Yao et al. 2015
**Dayingzhuang**<br>大营庄	Dianchi Lake, Kunming	2017	500	10	AMS<br>750-390<br>cal BC	35 pits<br>4 houses<br>5 rivers<br>2 floors<br>5 *jicao*	Pavillion structure		Dian type	*Oryza sativa*<br>*Setaria italica*<br>*Triticum aestivum*<br>*Hordeum vulgare*<br>*Chenopodium* sp.<br>*Zantoxhylum* sp.	n/a	Dal Martello 2020;<br>Dal Martello et al. 2021

Table S2. Comparison of sites in Yunnan with evidence for ancient plant remains.

Latin names indicate systematic environmental sampling and analysis, common English names indicate the remains were hand-picked or systematic analysis is still to be undertaken.

Site	Location	Exc. Date	Exc. Area m^2	Estimated site size ha	Chronology	Features	House structure	Burial type	Ceramics	Main archaeobotanical remains	Zooarchaeological remains	References
**Xueshan**<br>学山	Dianchi Lake, Chengjiang county	2010	35	1.4	c. 700-300 BC	20 houses<br>260+ graves<br>Unknown no. of pits	Semi-subterr./ wattle and daub	n/a	Dian type	*Triticum aestivum*<br>*Oryza sativa*<br>*Setaria italica*<br>*P. miliaceum*<br>*Glycine max*<br>*Fagopyrum* cf<br>*Hordeum vulgare*<br>Fruits<br>Acorns	*Margarya melanioides*	Wang 2014;<br>Wang et al. 2019
**Guangfentou**<br>光坟头	Fuxian Lake, Jiangchuan County	1984 2011/12	600	17	c. 700-300 BC	26 houses<br>30 pits<br>11 floors	Semi-cript		Dian type	*Triticum aestivum*<br>*Oryza sativa*<br>*Setaria italica*<br>*Hordeum* sp<br>*P. miliaceum*<br>*Chenopodium* sp.	n/a	Li and Liu 2016
**Xiaogucheng**	Dianchi Lake	2008 2010/11	Survey	n/a	c. 700-300 BC	n/a			Dian type	*Oryza sativa*<br>Panicoideae	n/a	Yao et al. 2015
**Shilinggang**<br>石岭岗	Middle Nujiang, Lushui county	2003<br>2013/14	500	10	AMS<br>723- 339<br>cal BC	42 graves<br>4 pits<br>4 floors<br>2 houses	wattle and daub?	shaft pits	Dianbian type	*Oryza sativa*<br>*Setaria italica*<br><br>[Isotopes]<br>Mix C3-C4;<br>Tubers, roots<br>Acorns<br>Palms	Pig<br>Goat<br>Cattle<br>Dog<br>Deer	Li et al. 2016;<br>Zhang et al. 2017;<br>Ren et al. 2017
**Yubeidi**<br>玉碑地	Bingu River (Jinsha River), Dongchuan County	2013	300	1.8	c. 700-200 BC	15 houses<br>49 pits<br>6 graves<br>Unknown no. of floors and postholes	Semi-subterr.	Urns	Dian type	*Oryza sativa*<br>*Triticum aestivum*<br>*Setaria italica*<br>*Glycine max*<br>*Chenopodium* sp.<br>*Zantoxhylum* sp.	n/a	Yang 2016;<br>Yang et al. 2020

Table S2. Comparison of sites in Yunnan with evidence for ancient plant remains.

Latin names indicate systematic environmental sampling and analysis, common English names indicate the remains were hand-picked or systematic analysis is still to be undertaken.

Site	Location	Exc. Date	Exc. Area m^2	Estimated site size ha	Chronology	Features	House structure	Burial type	Ceramics	Main archaeobotanical remains	Zooarchaeological remains	References
Jinlianshan 金莲山	Chengjiang	2008/2009	Over 600	n/a	700 BC	Over 260 burials		Shaft pits		[Isotopes] C3 prevalence (rice?); C4 secondary	n/a	Zhang 2011
Mayutian 麻玉田	Red River, Yuanjiang section	2006	n/a	n/a	c. 400-300 BC	16 burials		Vertical shaft pits		[Isotopes] Mixed C3-C4	n/a	Xiao and Wan 2013; Zhang et al. 2014
Qujing Dongjia Village 曲靖董家村	Dian Basin	1982	n/a	n/a	c. 700-300 BC	Cemetery		n/a		Rice	n/a	Li and Li 1983

References

An, Zhimin 安志敏 (1999). Zhongguo daozuo wenhua de qihua he dongchuan 中国稻作文化的起源和东传 (The origin and eastward spread or rice-planting culture of China). *Wenwu* 2: 63+70-92.

Chen, Jun 陈君, Quanjia Chen 陈全家 and Zhonghua Zhu 朱忠华 (2019). Yunnan Zongzan yizhi dongwu gugu yicun yanjiu 云南宗咱遗址出土动物骨骼遗存研究 (Research on the animal bone remains from the site of Zongzan, Yunnan). *Bianjiang Kaogu Yanjiu* 2:247-263..

Dal Martello, Rita (2020). *Agricultural Trajectories in Yunnan, Southwest China: a comparative analysis of archaeobotanical remains from the Neolithic to the Bronze Age.* Unpublished PhD thesis, UCL (University College London), London.

Dal Martello, Rita, Rui Min, Chris Stevens, Charles Higham, Thomas Higham, Ling Qin, and Dorian Q Fuller (2018). Early agriculture at the crossroads of China and Southeast Asia: archaeobotanical evidence and radiocarbon dates from Baiyangcun, Yunnan. *Journal of Archaeological Science: Reports* 20:711-721.

Dal Martello, Rita, Rui Min, Chris Stevens, Charles Higham, Thomas Higham, Ling Qin, and Dorian Q Fuller. 2018. Early agriculture at the crossroads of China and Southeast Asia: archaeobotanical evidence and radiocarbon dates from Baiyangcun, Yunnan. *Journal of Archaeological Science: Reports* 20:711-721.

He, J. (1990). Tonghai Haidong cun beiqiu yizhi 通海海东村贝丘遗址 (The shell-midden site of Haidong in Tonghai). In *Zhongguo Kaoguxue Nanjian*, 304-305. Beijing: Cultural Relics Press.

Jin, Hetian 金河天(2013). *Haimenkou yizhi zhiwu yicun zonghe yanjiu 海门口遗址植物遗存综合研究 (Comprehensive study on the archaeobotanical remains from Haimenkou*). Unpublished PhD thesis, Peking University, Beijing.

Jin, Hetian, Xu Liu, Min Rui, Xiaorui Li, and Xiaohong Wu (2014). Early subsistence practices at prehistoric Dadunzi in Yuanmou, Yunnan: new evidence for the origins of Early Agriculture in Southwest China. In *The 'Crescent-Shaped Cultural-Communication Belt': Tong Enzheng's Model in Retrospect. An Examination of Methodological, Theoretical and Material Concerns of Long-Distance Interactions in East Asia*. ed. Hein, A. 133-140. BAR International Series 2679, Oxford.

Kan, Y. 阚勇(1983). Yunnan Gengma Shifodong Yizhi Chutu Tanhua Gudao 云南耿马石佛洞遗址出土炭化古稻 (Charred rice discovered in shifodong site in Yunnan province). *Nongye Kaogu* 2:80-83.

Li, Hunsheng 李昆声, and Baolun Li 李保伦 (1983). Yunnan Qujing Faxian Tanhua Gudao 云南曲靖发现炭化古稻 (Ancient charred rice discovered at Qujing, Yunnan). *Nongye Kaogu* 2:78-79+72

Li, HaiMing, XinXin Zuo, LiHong Kang, LeLe Ren, FengWen Liu, HongGao Liu, NaiMeng Zhang, Rui Min, Xu Liu, and GuangHui Dong (2016). Prehistoric agriculture development in the Yunnan-Guizhou Plateau, southwest China: Archaeobotanical evidence. *Science China Earth Sciences* 59 (8):1562-1573.

Li, K, and R Min (2014). The site of Haimenkou: New research on the chronology of the Early Bronze Age in Yunnan. In The '*Crescent-Shaped Cultural-Communication Belt': Tong Enzheng's Model in Retrospect. An Examination of Methodological, Theoretical and Material Concerns of Long-Distance Interactions in East Asia*. ed. Hein A. 123-132. BAR International Series 2679, Oxford.

Li, Xiaorui 李小瑞 (2016). Minyishiweitian- Yunnan Gudai de Zhuyao Nongye Zuowu 民以食为天——云南古代的主要农作物(Food comes as the first: Staple crops in ancient Yunnan). https://mp.weixin.qq.com/s/DmWuz1-rsmAum7h5W44MRw. Accessed 03.02.2021.

Li, XR, 李小瑞 and X Liu 刘旭(2016). Yunnan Jiangchuan Guangfentou Yizhi Zhiwu Yicun Fuxuan Jieguo ji Fenxi 云南江川光坟头遗址植物遗存浮选结果及分析(An analysis on the carbonized seeds and fruits from Guangfentou site in Jiangchuan, Yunnan). *Nongye Kaogu* 3:20-27.

Liu, X, and Z Dai (2008). 3000 Nian qian de Xueju Shenghuo: Gengma Shifodong Yizhi 3000 年前的穴居生活：耿马石佛洞遗址 (Cave life from 3000 years ago: the site of Shifodong, Gengma). *Zhongguo Wenhua Yichan* 6:84-87.

Ren, Lele, Xin Li, Lihong Kang, Katherine Brunson, Honggao Liu, Weimiao Dong, Haiming Li, Rui Min, Xu Liu, and Guanghui Dong (2017). Human paleodiet and animal utilization strategies during the Bronze Age in northwest Yunnan Province, southwest China. *PloS one* 12 (5):e0177867.

Wang, Juan (2018). *A zooarchaeological study of the Haimenkou Site, Yunnan Province, China*. Archaeology of East Asia: BAR international series 2902.

Wang, Q. (2014). *Yunnan Dengjiang Xian Xueshan Yizhi Zhiwu yicun fenxi 云南登江县学山遗址植物遗存分析 (Analysis of the archaeobotanical remains found at Xueshan site, in Dengjiang county, Yunnan).* Unpublished MA thesis, Shandong University, Jilin.

Wang, Qi 王祁, Zhilong Jiang 蒋志龙, Wei Yang 杨薇 , and Xuexiang Chen 陈雪香 (2019). Yunnan Dengjiang Xueshan yizhi zhiwu yicun fuxuan jieguo ji chubu yanjiu 云南澄江学山遗址植物遗存浮选结果及初步研究 (Preliminary research and archaeobotanical results from Xueshan, Dengjian county, in Yunnan). *Zhongguo Nongye* 38 (2):3-11.

Xiang, AnQiang, Wenxu Zhang, Shaojin Li, and Jituo Wang (2015). Yunnan Baoshan Changning Dabin Yingpanshan Xinshiqi Yizhi Chutu Gudao yanjiu 云南保山昌宁达丙营盘山新石器遗址出土古稻研究 (Study on the ancient rice recovered from Yingpanshan, in Baoshan Changning Dabin, Yunnan). *Xuaxia Kaogu* 1:41-45.

Xiao, MH. 肖明华 (2001). Yunnan Kaogu Shulu 云南考古述略 (Yunnan Archaeology). *Kaogu* 12:1063-1075.

Xiao, Minhua 肖明华 , and Chang Wan 万杨 (2013). Yunnan gejiushi mayutian qingtong shidai mudi de fajue 云南个旧市麻玉田青铜时代墓葬的发掘 (The excavation of the Mayutian cemetery, in Gejiu, Yunnan). *Kaogu* 3:15-25.

Xue, Yining (2010). *Yunnan Jianchuan Haimenkou Yizhi Zhiwu Yicun Chubu Yanjiu 云南剑川海门口遗址植物遗存初步研究 (A preliminary investigation on the archaeobotanical material from the site of Haimenkou in Jianchuan County, Yunnan).* Unpublished MA Thesis, Peking University, Beijing.

Yang, W. 杨微 (2016). *Yunnan Hebosuo he Yubeidi yizhi zhiwu yicun fenxi 云南河泊所和玉碑地遗址植物遗存分析 (Analyses on the archaeobotanical remains from Hebosuo and Yubeidi).* Unpublished PhD thesis, Shandong University, Jinan.

Yang, W 杨薇, Zhilong Jiang 蒋志龙, and Xuexiang Chen 陈雪香 (2020). Yunnan Dongchuan Yubeidi Yizhi (2013 Niandu) zhiwu yicun fuxuan jieguo ji chubu fenxi 云南东川玉碑地遗址（2013 年度）植物遗存浮选结果及初步分析 (Preliminary analysis and archaeobotanical results from the 2013 campaign at Yubeidi, Dongchuan county, in Yunnan). *Zhongguo Nongye* 39 (1):3-11.

Yao, Alice (2010). Recent developments in the archaeology of southwestern China. *Journal of Archaeological Research* 18 (3):203-239.

Yao, Alice, Valentín Darré, Jiang Zhilong, Wengcheong Lam, and Yang Wei (2020). Bridging the time gap in the Bronze Age of Southeast Asia and Southwest China (long title). *Archaeological Research in Asia* 22:100189.

Yao, Alice, and Zhilong Jiang (2012). Discovering the elusive Bronze Age settlements of the 'Dian'kingdom, China. *Antiquity* 86 (332):353-363.

Yao, Alice, Zhilong Jiang, Xuexiang Chen, and Yin Liang (2015). Bronze age wetland/scapes: complex political formations in the humid subtropics of southwest China, 900–100 BC. *Journal of Anthropological Archaeology* 40:213-229.

YPICRA, Yunnan Provincial Institute of Cultural Relics and Archaeology 南省文物考古研究所 (2002). Yunnan Yongping Xinguang yizhi Fajue Baogao 云南永平新光遗址发掘报告 (Excavation Report of the site of Xinguang, Yongping, Yunnan). *Kaogu Xuebao* 2:203-204.

YPICRA, Yunnan Provincial Institute of Cultural Relics and Archaeology 南省文物考古研究所 (2003). Yunnan Yongren Caiyuanzi Mopandi Yizhi 2001 nian fajue baogao 云南永仁菜园子磨盘地遗址 2001 年发掘报告 (Report on the 2001 excavation campaign of the sites of Caiyuanzi and Mopandi in Yongren, Yunnan). *Kaogu Xuebao* 2:263-296.

YPICRA, Yunnan Provincial Institute of Cultural Relics and Archaeology, and University of Chicago (2019). Yunnan Jinning Hebosuo he Shangxihe yizhi qingtong shidai beiqiu yizhi shijue jianbao 云南晋宁河泊所和上西河遗址青铜时代贝丘遗址视觉简报 (Preliminary report on the excavation of the Bronze Age shell-midden sites of Hebosuo and Shangxihe in Jinning, Yunnan). *Jianghan Kaogu* 2:17-29.

YPICRA, Yunnan Provincial Institute of Cultural Relics and Archaeology 云南省文物考古研究所 (2017). Yunnan Tonghai Xingyi yizhi fajue 云南通海兴义遗址发掘 (The excavation of the site of Xingyi in Tonghai, Yunnan). http://www.kaogu.cn/cn/xccz/20170324/57600.html. Accessed 6 May 2019.

YPM, Yunnan Provincial Museum 云南省博物馆 (1958). Jianchuan Haimenkou Wenhua Yizhi Qingli Jianbao. *Kaogu Tongxun* 6:5-12.

YPM, Yunnan Provincial Museum 云南省博物馆 (1977). Yuanmou Dadunzi Xinshiqi Shidai Yizhi 云南大敦子新石器时代遗址 (The Neolithic site of Dadunzi in Yuanmou). *Kaogu Xuebao* 1:43-71.

YPM, Yunnan Provincial Museum 云南省博物馆 (1981). Yunnan Binchuan Baiyangcun yizhi 云南宾川白羊村遗址 (The site of Baiyangcun in Binchuan, Yunnan. *Kaogu Xuebao* 3:349-368.

YPM, Yunnan Provincial Museum 云南省博物馆(1963). Yunnan Jijing Shizhaishan Gumu disi fajue jianbao 云南寂静石寨山古墓发掘简报 (Preliminary report on the 4th excavation of the ancient cemetery of Shizhaishan, in Jijing, Yunnan). *Kaogu* 1:480-485+486-488.

Zhang, Chi, and Hsiao-chun Hung (2010). The emergence of agriculture in southern China. *Antiquity* 84 (323):11-25.

Zhang, Naimeng, Guanghui Dong, Xiaoyan Yang, Xinxin Zuo, Lihong Kang, Lele Ren, Honggao Liu et al. (2017). Diet reconstructed from an analysis of plant microfossils in human dental calculus from the Bronze Age site of Shilinggang, southwestern China. *Journal of Archaeological Science* 83:41-48. doi:https://doi.org/10.1016/j.jas.2017.06.010.

Zhang, Q. (2011). Yunnan Chengjiang xian Jinlianshan mudi chutu rengu wending tongweisu de chubu fenxi 云南澄江县金莲山墓地出土人骨稳定同位素的初步分析 (Prelimenary isotopic analysis of human skeletons recovered from Jinlianshan cemetery, Chengjiang county, Yunnan). *Kaogu* 1:3-30.

Zhang, Xingxiang, James Burton, Zhengyao Jin, Minghua Xiao, Anchuan Fan, and Jifeng Xu (2014). Isotope studies of human remains from Mayutian, Yunnan Province, China. *Journal of Archaeological Science* 50:414-419.

Zhao, Zhijun (2003). Yunnan Yongren Mopandi Xinshiqi Shidai yizhi chutu daogu yicun fenxi 云南永仁磨盘地新石器时代出土稻谷遗存分析(Preliminary analysis on the archaeobotanical remains from the site of Mopandi, Yongren, Yunnan). *Kaogu Xuebao* 4:294-296.

Zhao, Zhijun (2010). Shifodong yizhi zhiwu yicun fenxi baogao 石佛洞遗址植物遗存分析报告 (Report on the analysis of the plant remains from the Shifodong site). In *Gengma Shifodong*. Science Press, Beijing.
